# Impact of metabolic risk factors on colorectal cancer burden in China: a comprehensive analysis of trends from 1990 to 2021

**DOI:** 10.3389/fnut.2025.1694231

**Published:** 2026-01-08

**Authors:** Zhouwei Zhan, Hanchen Zheng, Chuying Chen, Jiami Yu, Lina Zheng, Chunkang Yang, Zengqing Guo, Bijuan Chen

**Affiliations:** 1 Department of Medical Oncology, Clinical Oncology School of Fujian Medical University, Fujian Cancer Hospital, Fuzhou, Fujian, China; 2 Department of Radiation Oncology, Clinical Oncology School of Fujian Medical University, Fujian Cancer Hospital, Fuzhou, Fujian, China; 3 Department of Gastrointestinal Surgery, Clinical Oncology School of Fujian Medical University, Fujian Cancer Hospital, Fuzhou, Fujian, China

**Keywords:** colorectal cancer, metabolic risks, high BMI, fasting plasma glucose, disease burden, China, Global Burden of Disease

## Abstract

**Background:**

Colorectal cancer (CRC) represents a significant public health challenge in China. With the rising prevalence of metabolic disorders such as obesity and diabetes, it is essential to understand the long-term burden of CRC attributable to metabolic risks.

**Methods:**

This study utilized data from the Global Burden of Disease (GBD) 2021 to estimate the burden of CRC attributable to metabolic risks in China from 1990 to 2021. Metrics analyzed included deaths, disability-adjusted life years (DALYs), years of life lost (YLLs), and years lived with disability (YLDs), stratified by age, sex, and year. Joinpoint regression, age-period-cohort analysis, and decomposition methods were employed to explore temporal trends and underlying drivers.

**Results:**

In 2021, CRC attributable to metabolic risks caused 36,462 deaths and 902,557 DALYs in China, with significantly higher burdens among men. The age-standardized death and DALY rates were 2.36 and 56.09 per 100,000 in men, respectively, nearly double those in women. Between 1990 and 2021, age-standardized rates of mortality, DALYs, YLDs, and YLLs increased significantly (AAPC > 0; *p* < 0.05), especially in males and older adults. High body mass index (BMI) showed a significantly steeper rise in ASRs than high fasting plasma glucose (HFPG) (*p* < 0.05), although HFPG exhibited smaller—and in some strata non-significant—changes (*p* ≥ 0.05). Compared to global trends, China experienced a sharper rise in CRC burden, particularly in YLDs. Joinpoint regression identified significant increases in mortality and DALYs (*p* < 0.05), with an acceleration after ~2007, while age-period-cohort analysis showed a pronounced increase in mortality and DALY rates among older age groups and more recent birth cohorts. Decomposition analysis indicated that in men, epidemiological changes primarily drove the increased burden, while in women, population aging and growth were dominant contributors.

**Conclusion:**

The burden of CRC attributable to metabolic risks has grown substantially in China, especially among men and older adults, with high BMI as the primary contributor. These findings highlight the urgent need for targeted prevention strategies addressing metabolic risk factors and sex-specific health interventions.

## Introduction

Colorectal cancer (CRC) is a major global public health concern, ranking among the top three most commonly diagnosed cancers and leading causes of cancer-related deaths worldwide (1, 2). In China, the burden of CRC has escalated significantly over the past three decades, driven by demographic shifts, lifestyle transitions, and the rapid pace of urbanization (3, 4). While early screening and therapeutic advances have helped reduce age-standardized mortality in some high-income countries, low- and middle-income countries such as China continue to face increasing absolute numbers of CRC cases and deaths (5, 6). Metabolic risk factors, such as elevated body mass index (BMI) and high fasting plasma glucose (HFPG), have become key drivers of the increasing disease burden, particularly amid the escalating prevalence of diabetes and obesity within the Chinese population (7–9).

Epidemiological studies have consistently identified metabolic dysregulation as a significant determinant of CRC incidence and progression (10). HFPG and type 2 diabetes are associated with chronic hyperinsulinemia and insulin resistance, conditions that may promote carcinogenesis through pro-inflammatory and mitogenic signaling pathways (11, 12). Likewise, high BMI is associated not only with insulin resistance but also with systemic inflammation, disrupted adipokine signaling, and imbalanced gut microbiota, all of which are implicated in the development of CRC (13–15). These overlapping mechanisms underscore the importance of understanding the metabolic underpinnings of CRC and quantifying their impact on disease burden to inform targeted prevention strategies.

Despite mounting evidence supporting the role of metabolic factors in CRC development, comprehensive assessments of their population-level burden in China remain scarce. Previous Global Burden of Disease (GBD) studies have quantified the health loss attributable to various metabolic risks globally and nationally, but specific estimates for CRC attributable to metabolic risks, stratified by age, sex, and time trends in China, are limited (16). Moreover, the evolving dynamics of this burden over the past three decades, particularly the respective contributions of population growth, population aging, and shifts in epidemiological patterns, remain incompletely understood. Using data from the GBD 2021 study, the present research aims to fill this gap by comprehensively analyzing the trends and drivers of CRC burden attributable to metabolic risk factors in China from 1990 to 2021, providing an evidence base to inform public health policymaking and resource allocation.

## Methods

### Data source

This study is a retrospective analysis based on data from the GBD 2021, which provides comprehensive and standardized estimates for 371 diseases and injuries and 88 risk factors across 204 countries and territories from 1990 to 2021. The GBD framework employs advanced statistical models and extensive data integration to ensure comparability across time and locations (17, 18). Specifically, the present study focused on colon and rectum cancer burden attributable to metabolic risks in China, including high BMI and HFPG. We obtained the estimates for the number of deaths, disability-adjusted life years (DALYs), years of life lost (YLLs), and years lived with disability (YLDs) from the Global Health Data Exchange (GHDx) query tool.^1^ For each metric, we extracted point estimates with 95% uncertainty intervals (UIs). Data were stratified by sex, 5-year age group, and year for the period 1990–2021. Age-standardized rates (ASRs) were calculated using the GBD standard population, enabling temporal comparisons independent of demographic structure.

The GBD methodology integrates data from vital registration systems, cancer registries, household surveys, hospital records, and other national surveillance systems. Disease burden attributable to each metabolic risk factor was estimated using a comparative risk assessment framework, incorporating relative risks derived from meta-analyses of epidemiological studies, exposure levels, and theoretical minimum risk exposure levels (TMRELs) (19, 20). The final estimates were generated using tools such as the Cause of Death Ensemble model (CODEm), Bayesian meta-regression (DisMod-MR 2.1), and spatiotemporal Gaussian process regression (ST-GPR) to model cause-specific mortality, non-fatal outcomes, and risk factor exposures. To address variability in data completeness and diagnostic coding across regions and years, the GBD pipeline applies standardized corrections (e.g., garbage-code redistribution, completeness adjustments, and internal cross-validation within ensemble models). Nevertheless, residual uncertainty may persist—particularly in earlier years and in under-resourced provinces—so absolute levels should be interpreted with caution, while long-term directional trends are comparatively more robust. Terminology was harmonized throughout the manuscript to use TMREL, in line with GBD 2021 documentation.

### Definition and estimation

In accordance with the GBD 2021 framework, CRC refers to malignant neoplasms of the colon and rectum, corresponding to ICD-10 codes C18-C21. The study focused on the burden of CRC attributable to two key modifiable metabolic risk factors: high BMI and HFPG, as defined by GBD criteria. High BMI for adults (aged ≥20 years) was defined as a BMI exceeding the theoretical minimum risk exposure level (TMREL) of 20–23 kg/m^2^, based on evidence indicating the lowest mortality risk within this range. For children and adolescents aged 2–19 years, high BMI was defined as overweight or obesity using the International Obesity Task Force (IOTF) age- and sex-specific standards. HFPG was defined as fasting plasma glucose levels greater than 4.9–5.3 mmol/L (TMREL range), which reflects the TMREL identified for metabolic risk factors in relation to CRC burden (17). All GBD estimates were extracted as point values with 95% UIs.

The attributable burden was estimated using the GBD comparative risk assessment framework. This approach involved determining the population-attributable fractions (PAFs) for each risk factor, representing the proportion of CRC burden that could be avoided if exposure were reduced to the TMREL. The estimation process integrated three elements: (1) population exposure distributions to high BMI and HFPG by age, sex, year, and location; (2) relative risk estimates derived from large-scale epidemiological studies and meta-analyses; and (3) disease burden estimates, including incidence, mortality, and disability. Modeling tools used included the DisMod-MR 2.1 Bayesian meta-regression for non-fatal outcomes, the CODEm for mortality, and ST-GPR for exposure estimation. DALYs were calculated as the sum of YLLs due to premature mortality and YLDs, providing a comprehensive measure of overall health loss attributable to metabolic risks. Consistent with GBD guidance, PAFs for high BMI and HFPG were estimated independently against their respective TMRELs; potential mediation or overlap between these correlated metabolic exposures was not modeled. To avoid double counting, risk-attributed burdens were not summed across risks, and comparisons are interpreted by metric (e.g., levels vs. trends) rather than as additive effects.

### Descriptive analysis

A descriptive analysis was conducted to assess the burden of CRC attributable to metabolic risks, specifically high BMI and HFPG, in China from 1990 to 2021. The analysis was stratified by sex, age group, and calendar year. The primary indicators included the absolute number and ASRs (per 100,000 population) of deaths, DALYs, YLDs, and YLLs. Age-standardization was performed using the GBD world standard population to allow for valid comparisons across time and between subgroups. To characterize the distribution in 2021, we examined the all-age number and ASRs of mortality, DALYs, YLLs, and YLDs by sex and age group. Sex- and age-specific patterns were visualized to highlight disparities and age-related trends in disease burden. We also compared the burden metrics between 1990 and 2021 to assess temporal changes in crude and standardized rates. The relative contributions of high BMI and HFPG to the total CRC burden were evaluated using attributable fractions and absolute burden estimates for each risk factor. Data visualization techniques were employed to depict temporal trends and demographic distributions using bar charts and line graphs. Supplementary figures were generated to illustrate differences in burden across age groups, sexes, and risk factors at both baseline and the most recent time point.

### Joinpoint regression analysis

Joinpoint regression analysis was conducted to evaluate temporal trends in ASRs of mortality, DALYs, YLDs, and YLLs for CRC attributable to metabolic risks in China from 1990 to 2021. This method identifies statistically significant changes in trend data over time by fitting a series of joined straight lines on a logarithmic scale and estimating the annual percentage change (APC) for each segment. The average annual percentage change (AAPC) was also calculated to provide an overall summary measure of trend across the entire period. The Joinpoint Regression Program (version 5.2.0) developed by the US National Cancer Institute was employed for this analysis. We evaluated models with up to five joinpoints and enforced a minimum segment length of ≥3 years to reduce overfitting. The optimal number and location of joinpoints were selected using Monte-Carlo permutation tests, with auxiliary fit checks via AIC/BIC that were concordant with the permutation-based choice. Statistical significance was set at a two-sided *p*-value < 0.05 (21). ASRs were log-transformed before model fitting to stabilize variances and accommodate heteroscedastic errors. Separate joinpoint models were fit for each outcome (mortality, DALYs, YLDs, YLLs), for each risk (high BMI, HFPG), and for both sexes combined and stratified by sex, allowing assessment of whether changes in CRC burden due to high BMI and HFPG differed between men and women over time. The regression results are presented as APCs and AAPCs with corresponding 95% confidence intervals (CIs), and the locations of significant joinpoints are illustrated graphically. Sensitivity analyses restricting the maximum to three joinpoints or permitting four to five joinpoints yielded comparable AAPC/APC estimates, with change-points typically within ±1–2 years.

### Age-period-cohort (APC) analysis

To investigate the temporal dynamics in CRC burden attributable to metabolic risks in China, an APC model was applied. This method separates observed trends into age, period, and cohort effects, clarifying how aging, historical context, and generational shifts shape disease burden. Given the linear dependency among the three temporal dimensions, the intrinsic estimator (IE) approach was utilized to generate consistent and statistically reliable estimates for each component (3, 22). Data on CRC-related mortality and DALYs from 1990 to 2021 were sourced from the GBD 2021 database and were categorized into five-year age brackets from 20 to 24 up to 90–94 years. Corresponding five-year periods (e.g., 1992–1996 to 2017–2021) and overlapping birth cohorts (e.g., 1900–1904 to 1997–2001) were generated accordingly. Individuals aged below 20 and above 94 were grouped at the respective extremes to maintain data robustness. Midpoints of age and period intervals were used to define cohort classifications. To adjust for demographic differences and enhance comparability, age-specific and period-specific standardized rates were used as outcome variables. The APC modeling process was performed using the “Epi” package (version 2.46) in R software (version 4.3.1). Model adequacy was confirmed through inspection of residual distributions and Akaike Information Criterion (AIC) values, ensuring optimal fit and interpretability of the estimated trends.

### Decomposition analysis

To quantify the driving forces behind the changes in the absolute number of CRC deaths and DALYs attributable to metabolic risks in China from 1990 to 2021, a decomposition analysis was conducted. This approach partitions the total change in disease burden into contributions from three distinct factors: population growth, population aging, and changes in epidemiological risk. In this study, “epidemiological rates” are defined precisely as the age-specific, risk-attributable rates (deaths or DALYs per age group attributed to high BMI or HFPG), thereby capturing shifts due to exposure distributions, relative risks, diagnosis/treatment, and other non-demographic influences, independent of population size and age structure. The decomposition method relies on a counterfactual framework to estimate the relative contribution of each factor by sequentially holding other components constant. Specifically, the total number of deaths and DALYs in 1990 was used as the reference scenario. Successive scenarios were constructed by modifying one component at a time: first updating population size while keeping age structure and disease rates constant, then adjusting the age distribution, and finally applying changes in age-specific rates to isolate epidemiological effects. The contributions of each factor were then calculated based on differences between these scenarios. This analysis was stratified by sex to assess gender-specific patterns in burden drivers.

## Results

### Burden of CRC attributable to metabolic risks in China, 2021

In 2021, CRC attributable to metabolic risks posed a considerable burden in China, with evident disparities across sexes and metrics. The total number of deaths attributable to these metabolic factors reached 36,462, with males accounting for a higher proportion than females. This pattern was consistently observed in all burden measures. Age-standardized death rates per 100,000 population were markedly higher among men (2.36) compared to women (1.32), indicating a greater mortality risk in the male population. In terms of DALYs, a cumulative total of approximately 902,557 years were lost due to premature death and disability, again predominantly affecting men. The age-standardized DALY rate was 56.09 per 100,000 in males and 30.66 in females, underscoring a substantial gender gap. While YLDs were lower in magnitude compared to YLLs, the male-to-female disparity persisted across both indicators. The majority of the burden was driven by YLLs rather than YLDs, emphasizing the fatal impact of metabolic-related CRC (Table 1).

**Table 1:** All-age cases and age-standardized deaths, DALYs, YLDs, and YLLs rates in 2021 for CRC attributable to metabolic risks in China.

Measure	All-ages cases (95% UI)			Age-standardized rates per 100,000 people (95% UI)		
	Total	Male	Female	Total	Male	Female
Deaths	36,462 (16,894, 57,808)	22,039 (9,791, 36,014)	14,423 (6,668, 23,465)	1.78 (0.82, 2.80)	2.36 (1.05, 3.84)	1.32 (0.61, 2.13)
DALYs	902,557 (417,029, 1,449,698)	565,219 (249,483, 933,120)	337,338 (159,277, 557,454)	42.87 (19.70, 68.63)	56.09 (24.70, 92.47)	30.66 (14.43, 50.55)
YLDs	43,888 (18,702, 70,758)	26,868 (11,395, 46,133)	17,020 (7,203, 28,861)	2.05 (0.87, 3.30)	2.61 (1.11, 4.45)	1.53 (0.64, 2.59)
YLLs	858,668 (395,682, 1,374,318)	538,350 (237,026, 887,322)	320,318 (150,143, 530,057)	40.83 (18.75, 65.23)	53.49 (23.51, 87.55)	29.14 (13.62, 48.05)

Values in parentheses indicate 95% UIs, estimated using Monte Carlo simulations. CRC, colon and rectum cancer; DALYs, disability-adjusted life-years; YLDs, years lived with disability; YLLs, years of life lost; UI, uncertainty interval.

### Age and sex patterns in CRC burden attributable to metabolic risks in China, 2021

**Figure 1:**
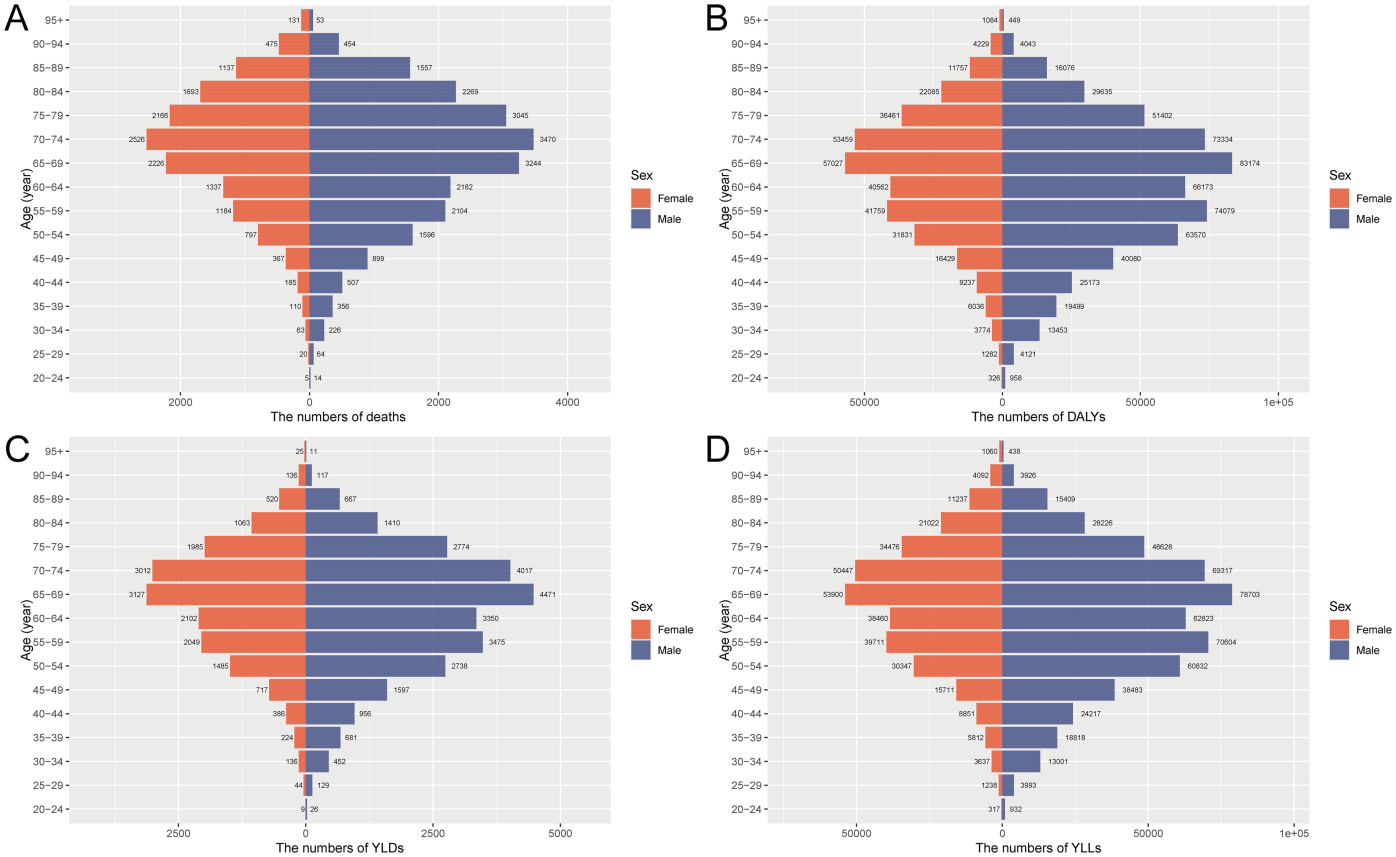
Sex- and age-specific numbers of deaths **(A)**, DALYs **(B)**, YLDs **(C)**, and YLLs **(D)** for CRC attributable to metabolic risks in China in 2021. DALY, disability-adjusted life year; YLD, years lived with disability; YLL, years of life lost; CRC, colorectal cancer.

**Figure 2:**
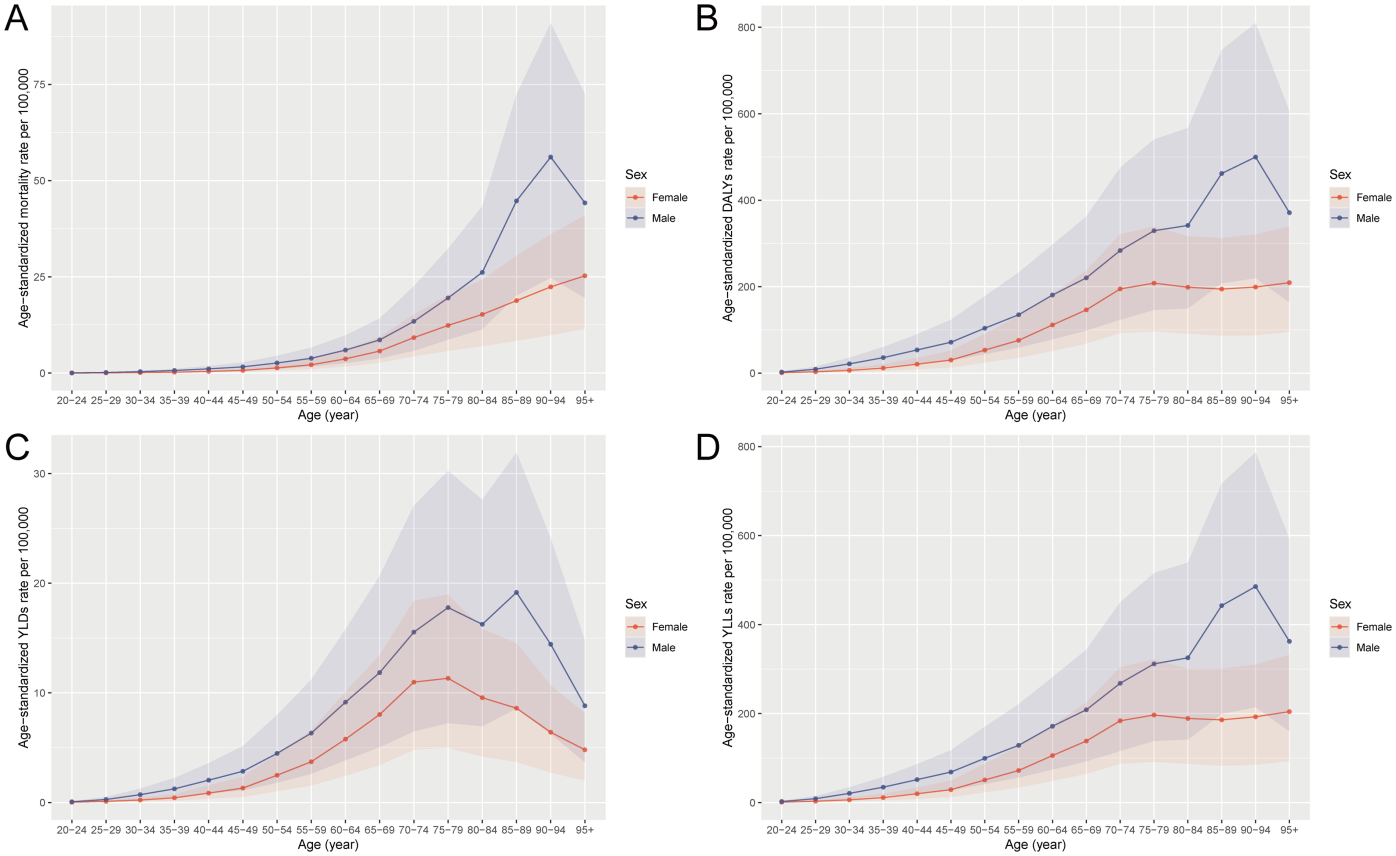
Age-specific and sex-specific trends in age-standardized rates of mortality **(A)**, DALYs **(B)**, YLDs **(C)**, and YLLs **(D)** for CRC attributable to metabolic risks in China in 2021. DALY, disability-adjusted life year; YLD, years lived with disability; YLL, years of life lost; CRC, colorectal cancer.

In 2021, the burden of CRC attributable to metabolic risks in China displayed distinct age and sex patterns across all metrics. The absolute numbers of deaths, DALYs, YLDs, and YLLs were substantially higher in males than females, with the burden intensifying markedly after the age of 50 and peaking in the 65–79 age range. While YLLs constituted the major component of DALYs, YLDs also increased steadily with age, reaching a peak in individuals aged 65–74 years (Figure 1). When standardized by age, mortality, DALY, YLD, and YLL rates all showed clear upward trends with increasing age, with males consistently exhibiting higher rates than females. The most pronounced age-related increases were observed for YLLs and DALYs, emphasizing the critical role of premature mortality in shaping the overall burden. These patterns underscore the disproportionate impact of metabolic risk factors on older adults and men in particular, highlighting the need for age- and sex-targeted interventions to mitigate CRC burden in the Chinese population (Figure 2).

### Temporal trends and age-specific changes in CRC burden attributable to metabolic risks in China, 1990–2021

**Figure 3:**
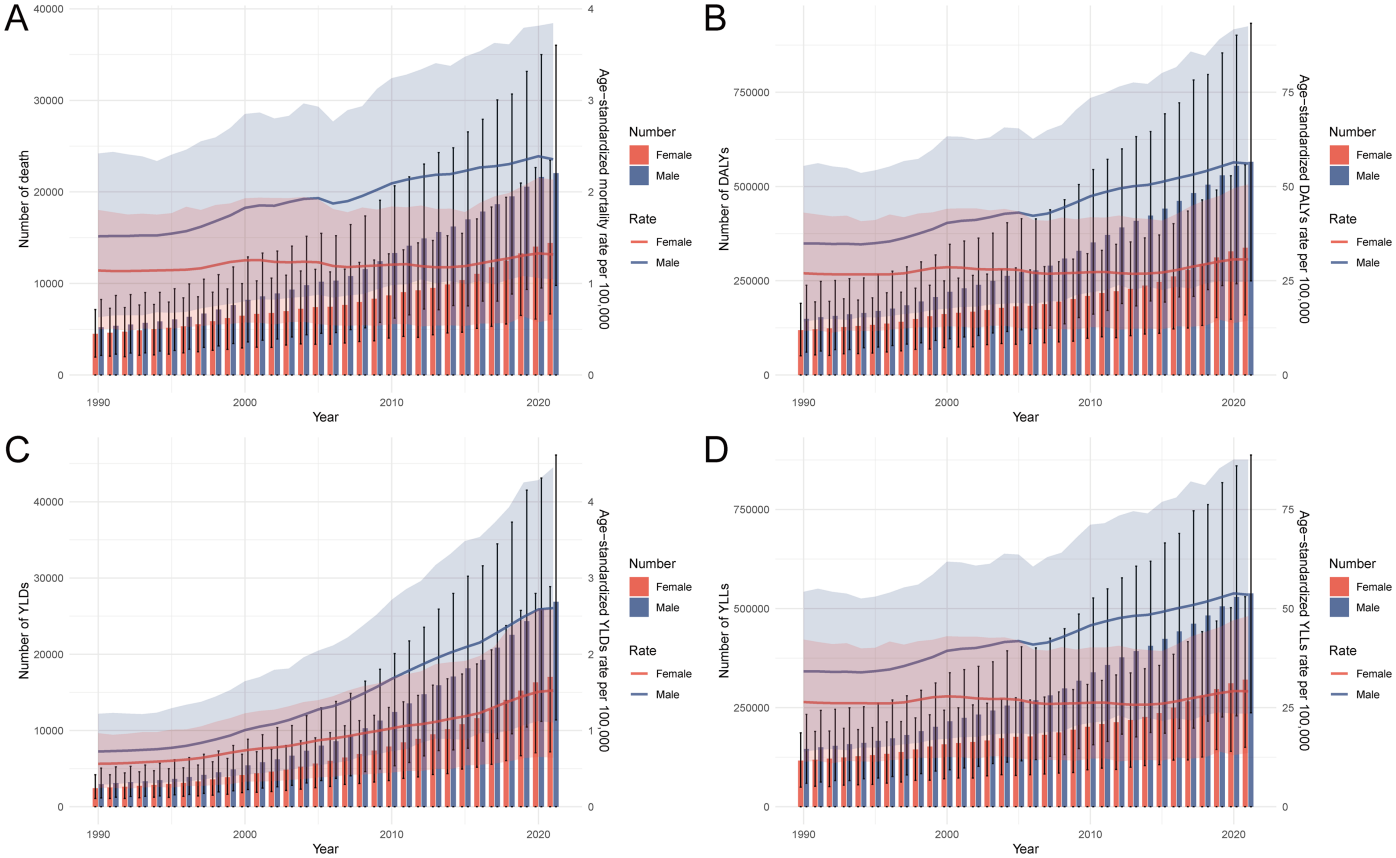
Temporal trends in numbers and age-standardized rates of deaths **(A)**, DALYs **(B)**, YLDs **(C)**, and YLLs **(D)** for CRC attributable to metabolic risks in China, 1990–2021. DALY, disability-adjusted life year; YLD, years lived with disability; YLL, years of life lost; CRC, colorectal cancer.

Between 1990 and 2021, the burden of CRC attributable to metabolic risks in China demonstrated substantial increases in absolute numbers of deaths, DALYs, YLDs, and YLLs, particularly among males (Figure 3). While ASRs of female mortality, DALYs, and YLLs remained relatively stable or showed only slight increases, these indicators exhibited more pronounced upward trends in males, reflecting widening sex disparities. Notably, the ASRs of YLDs continued to rise in both sexes, with a steeper increase observed in males, indicating a growing burden of disability associated with metabolic-related CRC. Supplementary analysis further revealed that, across all burden measures, both crude rates and absolute numbers escalated significantly from 1990 to 2021, especially among individuals aged 65 and older (Supplementary Figure S1). The most marked growth occurred in DALYs and YLLs, underscoring the increasing impact of premature mortality among older adults. These trends highlight the dual effects of demographic aging and persistent metabolic risk exposure, particularly in men, contributing to the escalating CRC burden in China.

### Contribution of specific metabolic risk factors to CRC burden in China, 1990–2021

In 2021, both high BMI and HFPG substantially contributed to the burden of CRC in China, with comparable absolute numbers of deaths and DALYs, and with HFPG showing a comparable level of YLLs relative to high BMI. According to Table 2 and Supplementary Table S1, high BMI-attributable CRC exhibited a marked increase in age-standardized mortality and DALY rates from 1990 to 2021, rising by 109.4 and 103.9%, respectively. In contrast, the corresponding rates for HFPG showed modest net change over the same period, increasing by 3.8% for mortality and 4.4% for DALYs. A similar trend was observed in YLLs and YLDs: high BMI led to substantial rises in age-standardized YLLs (98.3%) and YLDs (375.3%), while HFPG was associated with relatively stable YLLs (1.5%) and moderately increased YLDs (139.7%) (Supplementary Table S1). These patterns indicate a divergence whereby high BMI drives steeper long-term increases in ASRs, whereas HFPG attains a comparable YLL level in 2021. These differences were further visualized in Supplementary Figure S2, which showed steep upward trajectories for all ASRs associated with high BMI, whereas rates attributable to HFPG remained relatively flat.

**Table 2:** Mortality and DALYs for CRC attributable to metabolic risks in China, 2021, with trends in ASRs per 100,000 population, 1990–2021.

Metabolic risk	Deaths			DALYs		
	No, in thousands	Age-standardized rate per 100,000	Percentage change from 1990 to 2021	No, in thousands	Age-standardized rate per 100,000	Percentage change from 1990 to 2021
High body-mass index	19.4 (8.1, 32.5)	0.9 (0.4, 1.6)	109.4 (59.8, 196.7)	507.3 (209.3, 853.8)	24.2 (10.0, 40.7)	103.9 (54.8, 187.5)
High fasting plasma glucose	18.4 (9.2, 28.7)	0.9 (0.5, 1.4)	3.8 (−19.7, 33.2)	429.4 (210.5, 671.8)	20.2 (9.9, 31.6)	4.4 (−21.0, 36.0)

Values in parentheses indicate 95% UIs, estimated using Monte Carlo simulations. CRC, colon and rectum cancer; DALYs, disability-adjusted life years; ASRs, age-standardized rates; UI, uncertainty interval.

### Comparison of trends in CRC burden attributable to metabolic risks between China and global levels from 1990 to 2021

From 1990 to 2021, the burden of CRC attributable to metabolic risks in China exhibited significantly sharper increases across all age-standardized measures compared with the global average (Table 3 and Supplementary Figure S3). Specifically, the age-standardized mortality rate in China rose by 1.09-fold, from 1.3 to 1.78 per 100,000, a greater increase than the global change of 0.15-fold. Similarly, DALYs in China increased by 1.14-fold, while the global rise was only 0.21-fold. The most striking disparity was observed in YLDs, which surged nearly 3.87-fold in China compared to a 1.23-fold global increase, highlighting the growing survivorship burden. Age-standardized YLLs also rose by 1.05-fold in China, more than six times the global increase of 0.17-fold. These trends underscore the accelerating public health impact of metabolic risks on CRC in China, substantially outpacing global trajectories (Table 3). Supplementary Figure S3 clearly illustrates this disparity, showing a steeper upward trajectory in all indicators for China, particularly YLDs, compared to global trends.

**Table 3:** Change of age-standardized rates in deaths, DALYs, YLDs, and YLLs for CRC attributable to metabolic risks between 1990 and 2021 in China and global level.

Measure	China			Global		
	1990	2021	Change	1990	2021	Change
Deaths	1.3 (0.58, 2)	1.78 (0.82, 2.8)	1.09 (1.02–1.15)^*^	1.96 (0.9, 3.02)	2.05 (0.96, 3.09)	0.15 (0.13–0.18)^*^
DALYs	30.62 (13.71, 46.92)	42.87 (19.7, 68.63)	1.14 (1.10–1.19)^*^	42.54 (19.28, 65.58)	45.43 (21.4, 68.73)	0.21 (0.19–0.23)^*^
YLDs	0.64 (0.27, 1.07)	2.05 (0.87, 3.3)	3.87 (3.80–3.94)^*^	1.45 (0.64, 2.33)	2.12 (0.96, 3.4)	1.23 (1.20–1.25)^*^
YLLs	29.99 (13.37, 46.06)	40.83 (18.75, 65.23)	1.05 (1.01–1.10)^*^	41.09 (18.66, 63.55)	43.31 (20.36, 65.81)	0.17 (0.15–0.19)^*^

Values in parentheses indicate 95% UIs, estimated using Monte Carlo simulations. CRC, colon and rectum cancer; DALYs, disability-adjusted life-years; YLDs, years lived with disability; YLLs, years of life lost; UI, uncertainty interval; ^*^p < 0.05.

### Joinpoint regression and temporal trends in CRC burden attributable to metabolic risk factors in China from 1990 to 2021

**Figure 4:**
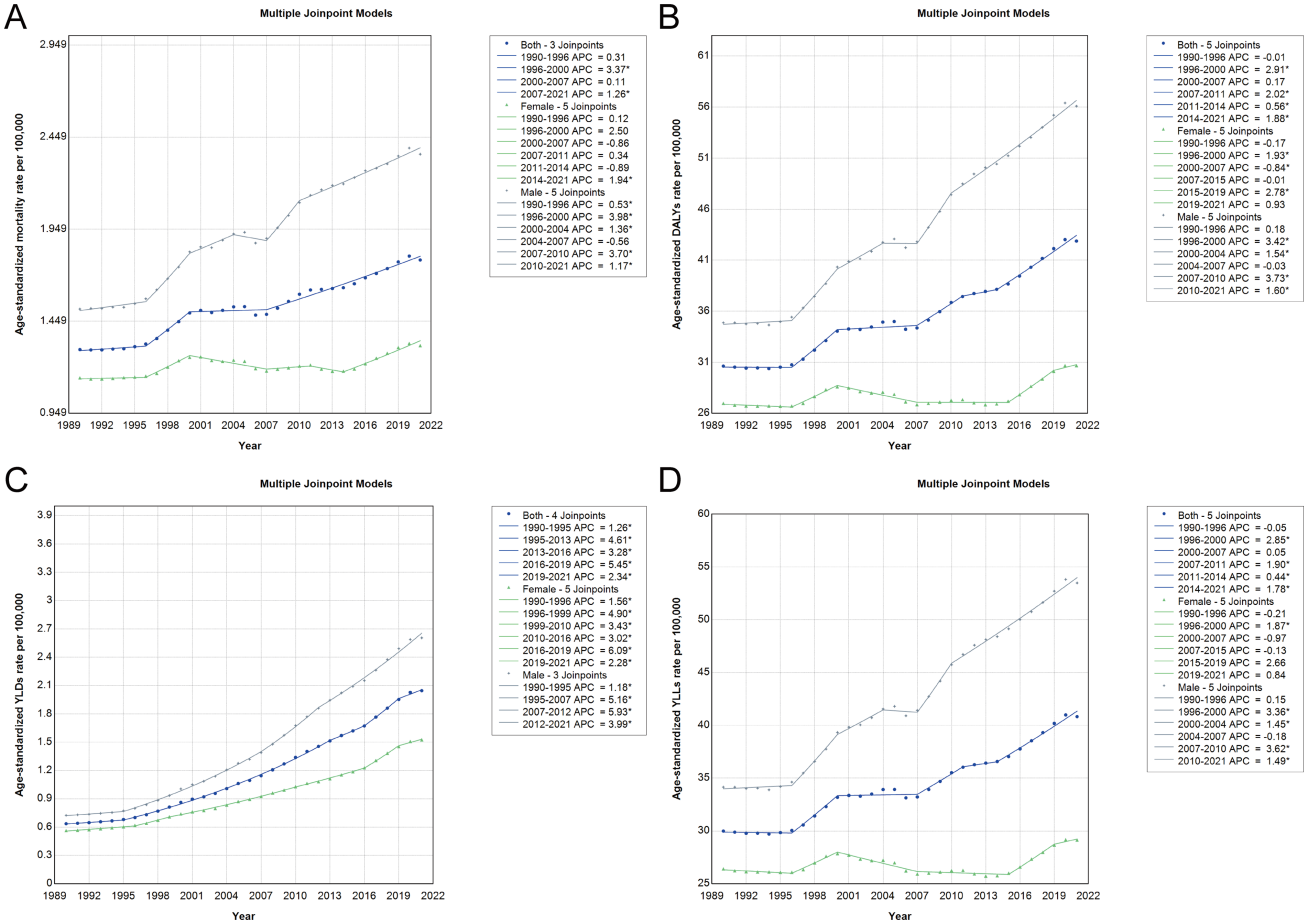
Joinpoint regression analysis of age-standardized rates for CRC attributable to metabolic risks in China, 1990–2021. **(A)** Trends in age-standardized mortality rates with APC estimates and identified joinpoints. **(B)** Trends in age-standardized DALY rates with APCs and joinpoints. **(C)** Trends in age-standardized YLD rates with APCs and joinpoints. **(D)** Trends in age-standardized YLL rates with APCs and joinpoints. CRC, colorectal cancer; APC, annual percentage change; DALY, disability-adjusted life year; YLD, years lived with disability; YLL, years of life lost.

Joinpoint regression analysis revealed dynamic temporal changes in the burden of CRC attributable to metabolic risks in China from 1990 to 2021. As shown in Figure 4 and detailed in Supplementary Table S2, age-standardized mortality and DALY rates exhibited a significantly increasing trend, with AAPCs of 1.09 and 1.14%, respectively, across the entire period. Both indices experienced multiple joinpoints, with particularly rapid increases observed between 1996–2000 and 2007–2021. Notably, the AAPC of mortality in males (1.50%) was nearly threefold higher than in females (0.54%), indicating a sex-specific disparity in risk trends. For YLDs, a more pronounced rise was detected, with AAPCs of 3.87% in both sexes, 4.29% in males, and 3.31% in females. The most substantial acceleration occurred in males from 2007 to 2012 (APC = 5.93%), aligning with a parallel increase in YLLs during the same period. Although some phases exhibited temporary stagnation or slight declines, especially among females in the early 2000s, the overall burden continued to escalate. These findings highlight a sustained upward trajectory in metabolic risk-attributable CRC burden, particularly among men, emphasizing the need for targeted intervention strategies (Figure 4 and Supplementary Table S2).

### Age-period-cohort effects on mortality and DALY rates of CRC attributable to metabolic risk factors in China

**Figure 5:**
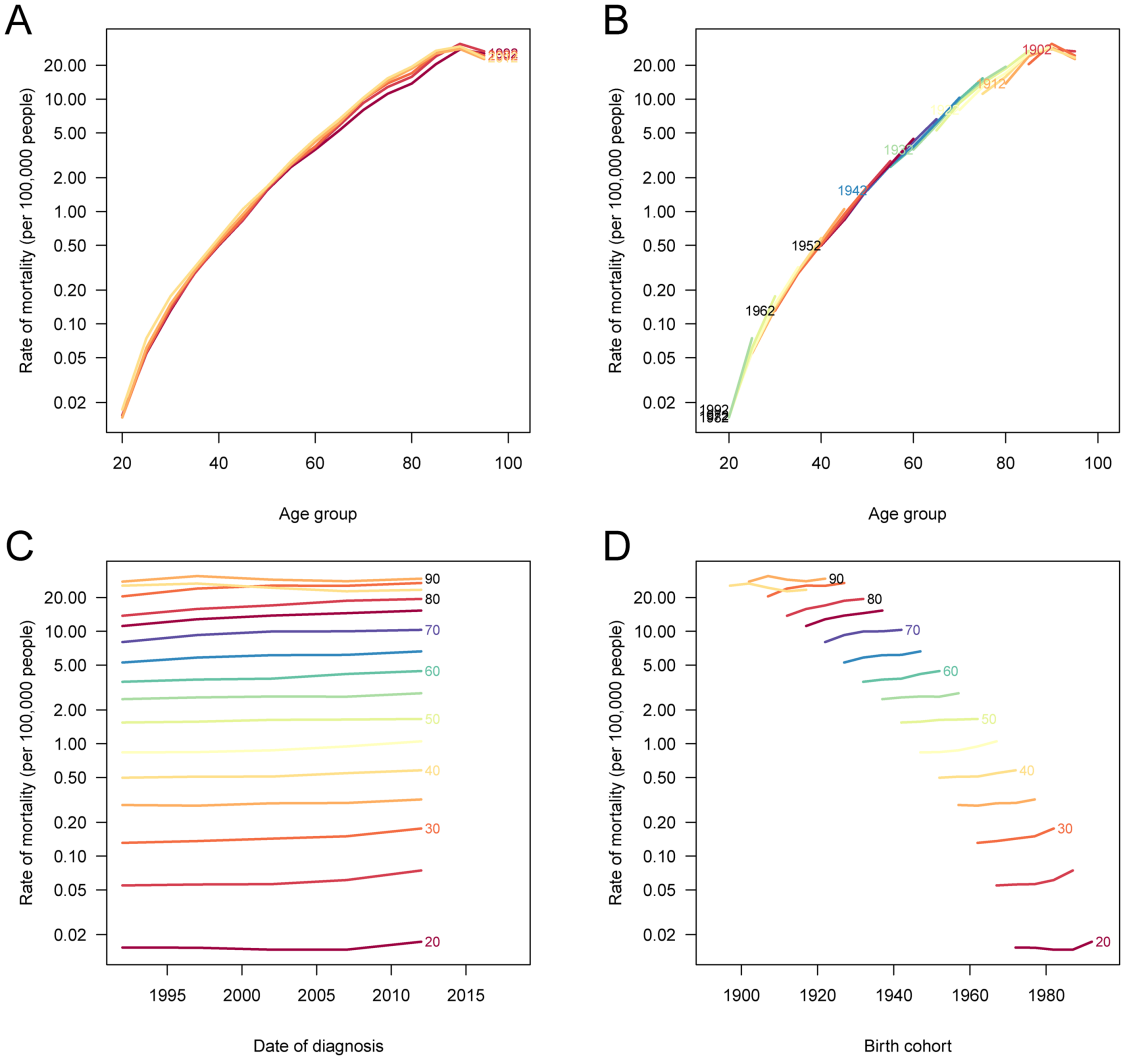
Age-period-cohort analysis of CRC mortality attributable to metabolic risks in China, 1990–2021. **(A)** The age-specific mortality rates according to time periods; each line connects the age-specific rates for a 5-year period. **(B)** The age-specific mortality rates according to birth cohorts; each line connects the age-specific rates for a 5-year cohort. **(C)** The period-specific mortality rates according to age groups; each line connects the period-specific rates for a 5-year age group. **(D)** The birth cohort-specific mortality rates according to age groups; each line connects the cohort-specific rates for a 5-year age group. CRC, colorectal cancer.

The age-period-cohort analysis offered further insights into the temporal patterns of CRC burden attributable to metabolic risks in China (Figure 5 and Supplementary Figure S4). Age-specific mortality and DALY rates increased steeply with advancing age, peaking among individuals aged 80–89 years across all time periods. Period-specific trends showed a steady rise in mortality and DALY rates from 1990 to 2021, especially among older age groups. Birth cohort-specific patterns revealed a consistent decline in rates among successive generations born before the 1960s. However, a concerning upward trend was observed in the more recent cohorts born after 1970, indicating an increasing cancer burden in younger populations likely due to the rising prevalence of obesity, diabetes, and other metabolic disorders. These cohort effects were more pronounced in mortality than DALY rates, underscoring the long-term impact of early-life metabolic exposures.

### Decomposition of changes in mortality and DALY rates of CRC attributable to metabolic risk factors in China (1990–2021)

**Figure 6:**
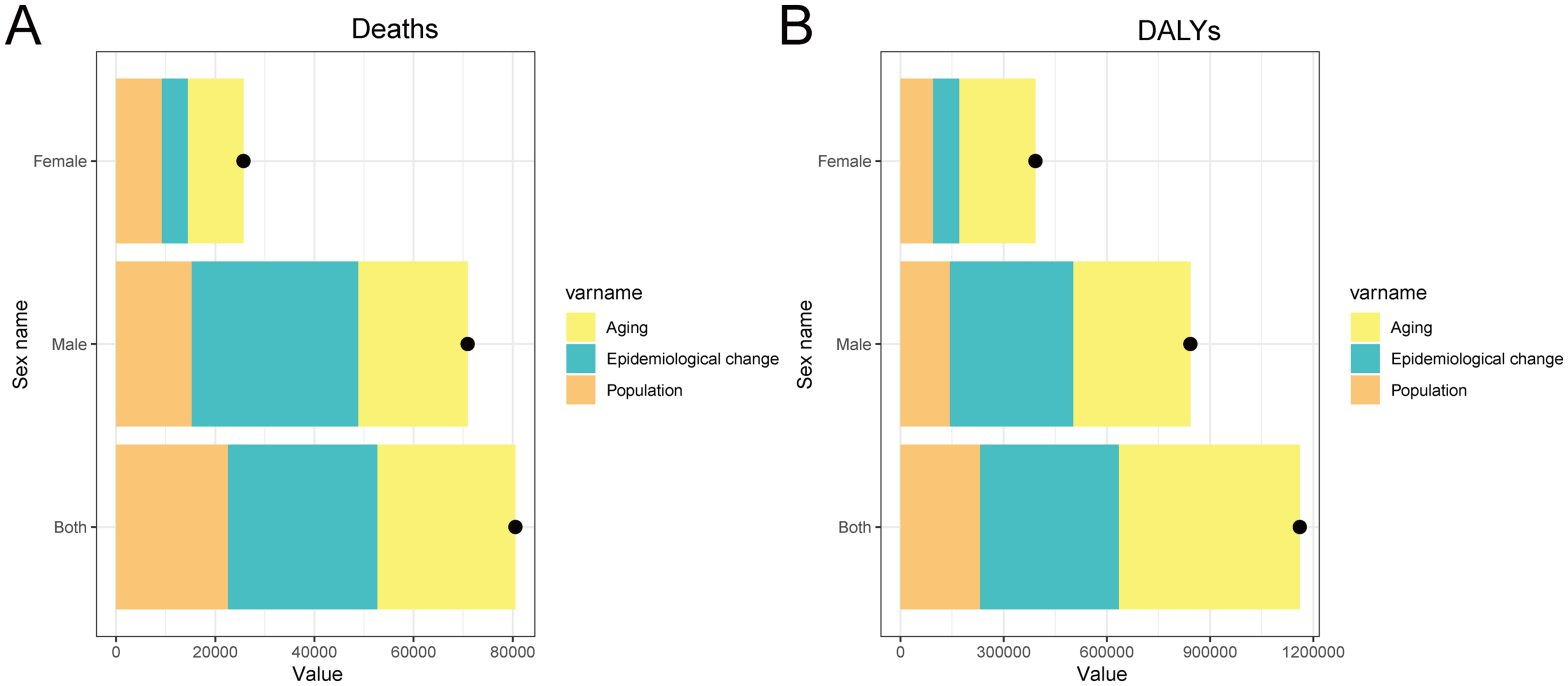
Decomposition analysis of changes in deaths **(A)** and DALYs **(B)** for CRC attributable to metabolic risks in China from 1990 to 2021, stratified by sex. DALY, disability-adjusted life year; CRC, colorectal cancer.

The decomposition analysis showed that for the general population, the overall increase in deaths and DALYs from CRC attributable to metabolic risks in China between 1990 and 2021 was primarily driven by population aging and population growth, with marked gender differences (Figure 6). In men, epidemiological changes were the main contributor to the increase in both deaths and DALYs, followed by population aging, while the contribution of population growth was relatively modest. In contrast, for women, population aging played the dominant role, followed by population growth, whereas the effect of epidemiological changes was comparatively minor. These findings highlight the differential drivers of disease burden across sexes and underscore the importance of implementing gender-sensitive strategies in cancer prevention and metabolic risk management.

## Discussion

This study offers a comprehensive analysis of the long-term trends in CRC burden attributable to metabolic risk factors in China from 1990 to 2021. Using GBD 2021 data, we observed that the absolute numbers of CRC-related deaths and DALYs attributable to metabolic risks have risen markedly over the past three decades, with a disproportionate increase among men. Despite age-standardized mortality and DALY rates remaining relatively stable or slightly increasing overall, a consistent upward trend in YLD rates was evident, particularly among males. Joinpoint regression revealed that the most recent periods experienced a noticeable uptick in disease burden, especially after 2006. Decomposition analysis demonstrated that population aging was the dominant driver of burden growth in women, whereas in men, epidemiological changes contributed more significantly. Age-period-cohort analysis further highlighted strong birth cohort effects, with individuals born more recently exhibiting increasing CRC burden, especially in males. Furthermore, our disaggregated analysis of metabolic risk factors showed that HFPG had a greater impact than high BMI across all burden metrics. These findings underscore the escalating public health challenge posed by metabolic risk-associated CRC and the urgent need for targeted interventions.

Our findings align with and extend several recent global studies on CRC attributable to metabolic risks, while providing new insights specific to the Chinese population. Prior studies such as those by He et al. (23) and Zhang et al. (24) emphasized the rising global burden of CRC attributable to high BMI and HFPG, noting a 2.4–2.47-fold increase in mortality from 1990 to 2021 and projecting continuing growth through 2046. However, those studies primarily focused on global or regional patterns, without detailing the heterogeneity within countries. Our study not only confirms a rising burden in China but also demonstrates how epidemiological transitions differ by sex and are strongly influenced by birth cohort effects. Furthermore, while Zhao et al. (25) and Zhang and Yang (26) discussed the role of sociodemographic index (SDI) and sex disparities in CRC burden, our decomposition analysis provided a more detailed attribution of burden changes to population aging, growth, and epidemiological shifts. This allows for more actionable national policy insights. By incorporating both high BMI and HFPG in our modeling and highlighting the pronounced increase among recent birth cohorts in China, our study fills important gaps left by earlier global projections and underscores the urgency of targeted metabolic risk mitigation strategies at the national level.

Compared with previous literature, our study provides a more nuanced perspective on the evolving burden of CRC attributable to metabolic risks in China by incorporating age-period-cohort and decomposition analyses. For example, Zhao et al. primarily quantified the global CRC burden associated with high BMI and projected trends to 2046 but did not differentiate the contributions of demographic versus epidemiological drivers (25). In contrast, our decomposition analysis revealed striking sex-specific differences, with epidemiological changes being the dominant driver in men, while population aging had a greater influence in women. Our findings further show that post-1970 birth cohorts exhibit steeper increases in mortality and DALYs, suggesting a cohort effect that aligns with earlier-life obesogenic exposures (dietary westernization, reduced physical activity, rising adolescent adiposity) and the global concern over early-onset CRC (27, 28). To contextualize this cohort signal with Chinese data, we note nationally reported increases in sugar-sweetened beverage consumption among youth (29), growth in recreational screen time with declines in physical activity (30), and rising adult adiposity/dysglycemia (31)—patterns consistent with metabolic pathways implicated in CRC. By analyzing high BMI alongside HFPG—and distinguishing trend (steeper long-term ASR/DALY increases for BMI) from level (HFPG showing comparable YLLs in 2021)—we provide a broader, internally consistent view of metabolic contributors. This comparative insight underscores the need for refined, data-driven policies targeted by metric (trend vs. level), sex, and cohort, and implemented via integrated weight-management and diabetes-control programs with risk-adapted FIT-based CRC screening.

Accumulating evidence supports the biological plausibility that metabolic risk factors, particularly HFPG and high BMI, play important roles in the development of CRC (32–35). Chronic hyperglycemia can promote cancer development through multiple mechanisms, including oxidative stress, pro-inflammatory cytokine release, and enhanced production of advanced glycation end products, which contribute to DNA damage and impaired apoptosis (36, 37). Insulin resistance, a common feature of obesity and type 2 diabetes, leads to compensatory hyperinsulinemia, which may stimulate cellular proliferation by activating the insulin-like growth factor-1 (IGF-1) axis (38, 39). In addition, adipose tissue in obese individuals secretes various adipokines, such as leptin and adiponectin, that can modulate inflammatory pathways and influence tumor behavior (40, 41). Experimental studies have shown that high-glucose conditions enhance colon epithelial cell proliferation and invasiveness, supporting a direct oncogenic role for glucose dysregulation (42, 43). Moreover, elevated plasma glucose levels may impair immune surveillance and promote tumor immune evasion. Importantly, many of these pathways (e.g., insulin/IGF-1 signaling, chronic inflammation, and adipokine imbalance) are shared between HFPG and high BMI, suggesting partial mediation and biological overlap rather than entirely independent mechanisms. Given that our epidemiologic analyses are ecological and attribution follows the GBD comparative risk assessment framework, these mechanistic links should be interpreted as supportive of plausibility rather than proof of causality at the individual level.

The observed differences in disease burden across sex, drivers, and birth cohorts likely reflect a complex interplay of biological, behavioral, and societal factors. Men consistently exhibited higher age-standardized mortality, DALY, and YLL rates attributable to metabolic risks compared to women, aligning with previous research suggesting a greater susceptibility among males to metabolic dysfunction and colorectal tumorigenesis (44). This disparity may be partially explained by sex-specific hormonal influences, such as the protective effects of estrogen in premenopausal women, and differential fat distribution patterns that modulate insulin sensitivity and inflammation (45). In terms of driving factors, decomposition analysis revealed that population aging and epidemiological changes contributed differently by sex; men were more affected by unfavorable epidemiological transitions, possibly linked to higher prevalence of obesity, poor glycemic control, and unhealthy lifestyle behaviors such as smoking and alcohol consumption (8, 46, 47). Notably, the upward trend in disease burden among recent birth cohorts implies increased early-life exposure to obesogenic environments, westernized diets, and sedentary behaviors, all of which are known to amplify long-term metabolic risk (48–50). These generational differences underscore the importance of life-course approaches to prevention, targeting modifiable risk factors beginning in childhood and adolescence to reverse unfavorable cohort trajectories.

The findings carry important implications for clinical practice and public health policy. Given the rising burden of colorectal cancer linked to metabolic risks, targeted interventions to improve metabolic health may offer considerable preventive benefits. Clinically, routine screening for glucose intolerance and obesity should be integrated into CRC risk assessment, especially for middle-aged men and those with metabolic family histories. In practice, this includes primary-care BMI and fasting-glucose checks tied to electronic prompts, brief lifestyle counseling, and referral pathways for weight management and diabetes prevention/management. From a policy perspective, strategies must extend beyond individuals to population-wide measures—healthy diets, limits on sugar-sweetened beverages, physical-activity infrastructure, and fiscal policies discouraging obesogenic behaviors. In China, these priorities can be embedded within Healthy China 2030 and the “Three Reductions, Three Health” campaign, alongside school/workplace programs, front-of-pack guidance, and food-service reformulation targets. For diabetes control, scaling structured lifestyle intervention and stepwise pharmacotherapy in community health centers—supported by family-doctor contract services—can address HFPG-linked premature mortality. On cancer control, expand risk-adapted CRC screening using FIT with colonoscopic follow-up and patient navigation, prioritizing high-incidence urban districts and integrating metabolic-risk counseling at screening. Given urban–rural and east–west heterogeneity, subnational tailoring with performance monitoring and periodic data-quality audits is warranted. Pairing weight-management policies to blunt BMI-driven ASR increases with strengthened diabetes control to reduce HFPG-linked YLLs—while embedding FIT-based screening in primary care—offers near-term, measurable gains.

Despite the comprehensive scope and robust methodology, several limitations warrant consideration. First, GBD-derived estimates integrate multiple sources and models and may carry uncertainty, particularly in China where registry coverage and data infrastructure evolved over three decades; urban–rural and east–west differences in ascertainment, coding, and completeness can yield regional heterogeneity. Although GBD applies garbage-code redistribution, completeness adjustments, and ensemble modeling, residual under-ascertainment and misclassification may remain—especially earlier and in under-resourced provinces. Moreover, we lacked permission to access restricted micro-/meso-level datasets from the National Cancer Registry Center or provincial registries; external checks therefore relied on publicly available aggregates and literature summaries, supporting trend-level (not absolute) concordance. Second, while we disaggregate burdens for high BMI and HFPG, interactions among metabolic factors and lifestyle confounders (diet, smoking, alcohol) are not modeled, and CRA cannot fully resolve mediation/overlap among correlated exposures. Consistent with GBD guidance, risk-specific PAFs were estimated independently against TMRELs and were not summed across risks to avoid double counting. Third, the ecological design limits causal inference at the individual level. Fourth, decomposition operates on point estimates without propagating GBD uncertainty intervals; percentage contributions are indicative magnitudes rather than probabilistic estimates. Fifth, national-level reporting (without province-level estimates or colon vs. rectum separation) may mask subnational or site-specific patterns relevant to targeting. Finally, sex-specific biological and behavioral determinants of observed disparities could not be fully explored. Future work should integrate individual-level longitudinal data, molecular profiling, and lifestyle information; strengthen subnational surveillance with periodic data-quality audits in underrepresented regions; and advance cohort studies while incorporating machine-learning tools into epidemiological models to refine risk prediction and inform precision prevention tailored to demographic and metabolic profiles.

## Conclusion

This study provides a comprehensive assessment of the long-term burden of CRC attributable to metabolic risk factors in China, offering valuable insights into the temporal dynamics and demographic patterns associated with these risks. The integration of multiple analytical approaches highlights the complex interplay between population aging, epidemiological transitions, and birth cohort effects. These findings underscore the pressing need for sustained public health efforts focused on metabolic health, lifestyle modification, and early detection strategies. In particular, our results indicate that high BMI drives steeper long-term increases in ASRs, whereas HFPG contributes a comparable level of YLLs in 2021, guiding distinct but complementary priorities. Future research should prioritize identifying high-risk populations through precision prevention, exploring the molecular mechanisms linking metabolic dysfunction to carcinogenesis, and evaluating the impact of public health interventions over time. From an implementation standpoint, policy actions with near-term feasibility include scaling weight-management programs to blunt BMI-driven trends, strengthening diabetes prevention and control pathways in primary care to reduce HFPG-linked premature mortality, and expanding risk-adapted FIT-based CRC screening with colonoscopic follow-up and navigation. In addition, developing sex-specific and age-targeted prevention frameworks and enhancing surveillance systems will be essential to curb the growing burden. Subnational tailoring (urban–rural/east–west) with performance monitoring and periodic data-quality audits can focus resources where gaps are greatest. Continued interdisciplinary collaboration between epidemiologists, clinicians, and policy makers is crucial to inform evidence-based strategies and reduce the preventable impact of metabolic risk factors on CRC.

## Data Availability

The datasets presented in this study can be found in online repositories. The names of the repository/repositories and accession number(s) can be found at: https://ghdx.healthdata.org/gbd-results-tool.

## Footnotes

1
https://vizhub.healthdata.org/gbd-results/
