# Supplementary figures and images for: Impact of metabolic risk factors on colorectal cancer burden in China: a comprehensive analysis of trends from 1990 to 2021
Source: Front Nutr. 2026 Jan 8;12:1694231. doi: 10.3389/fnut.2025.1694231 (PMC12824012; doi:10.3389/fnut.2025.1694231)

A

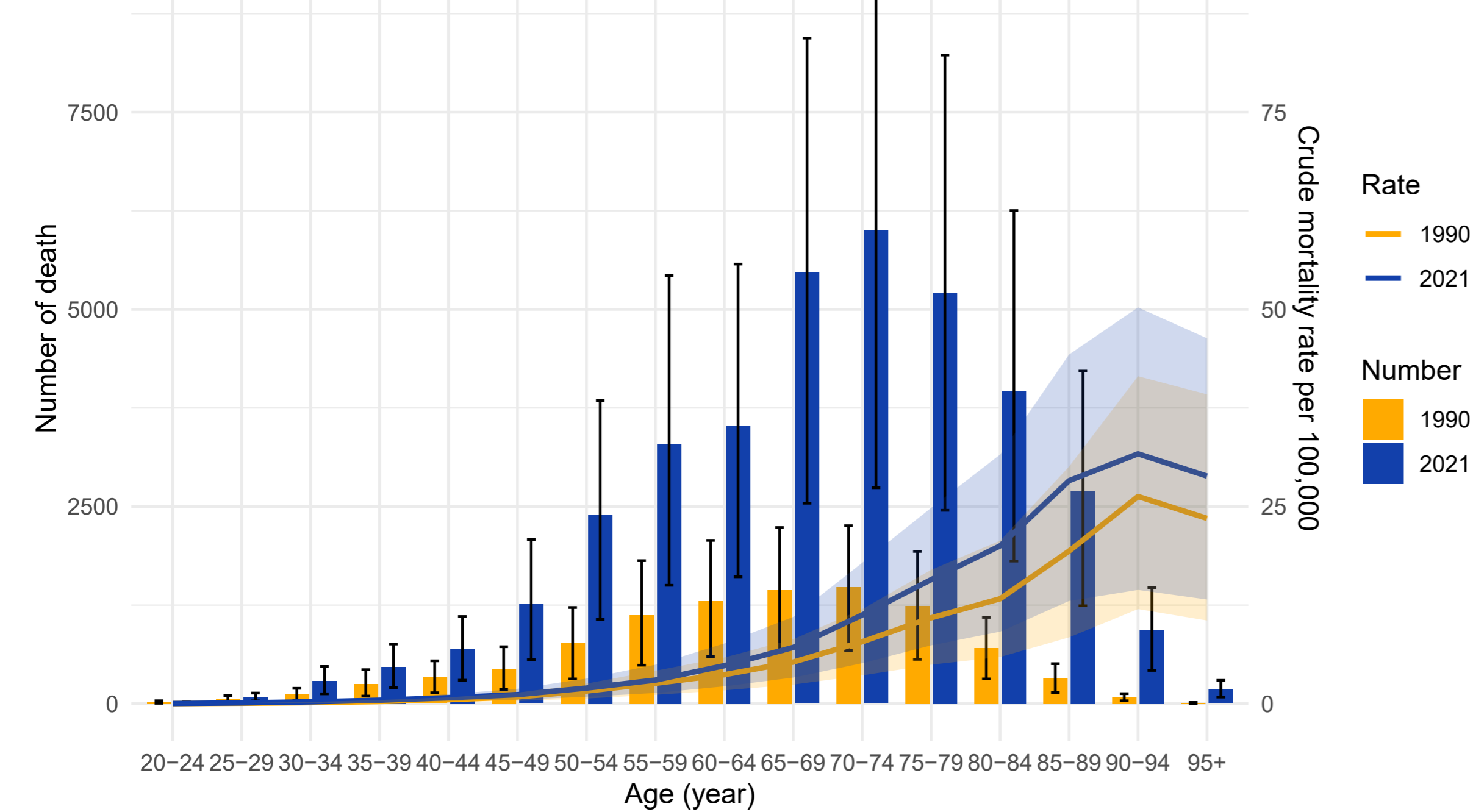

B

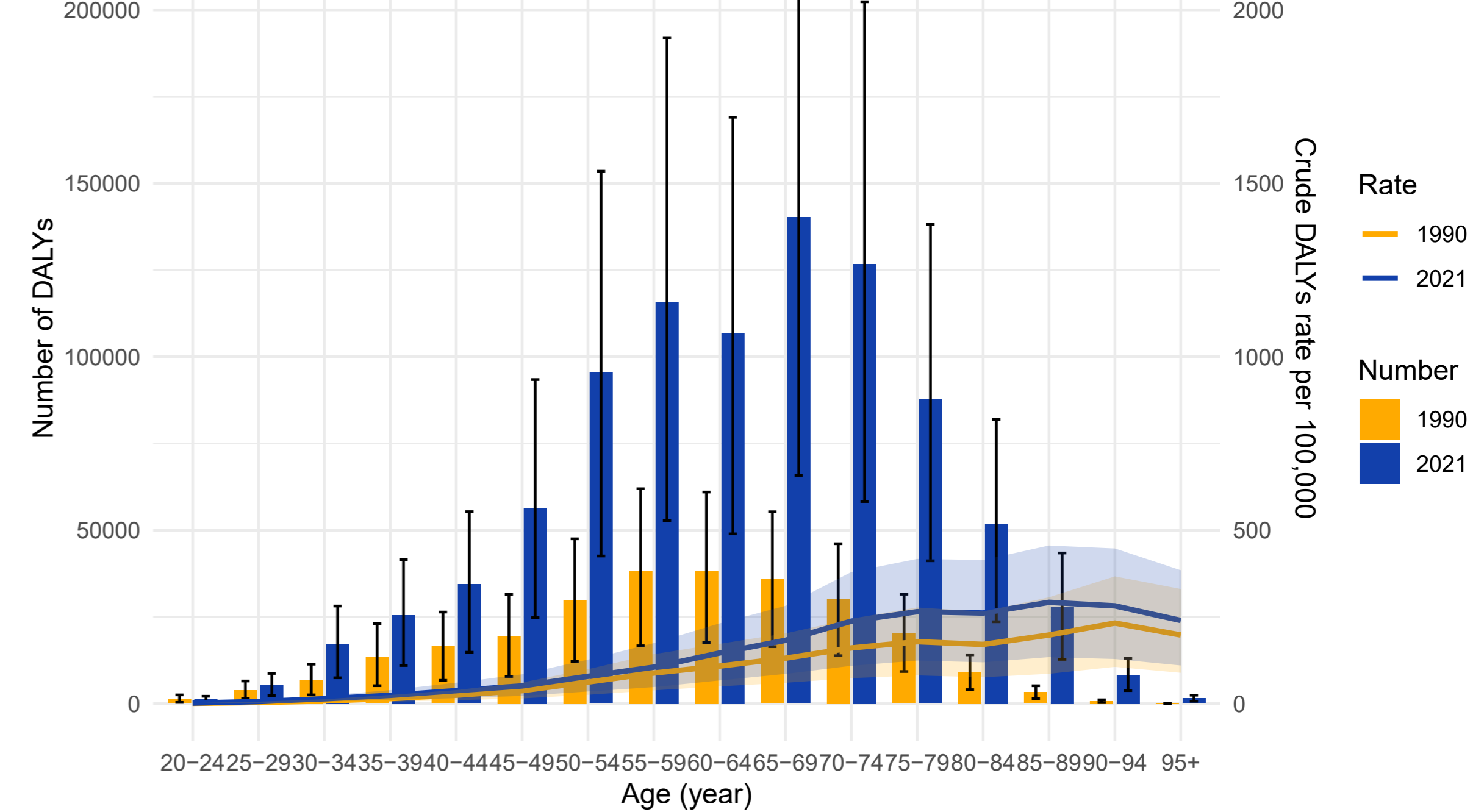

C

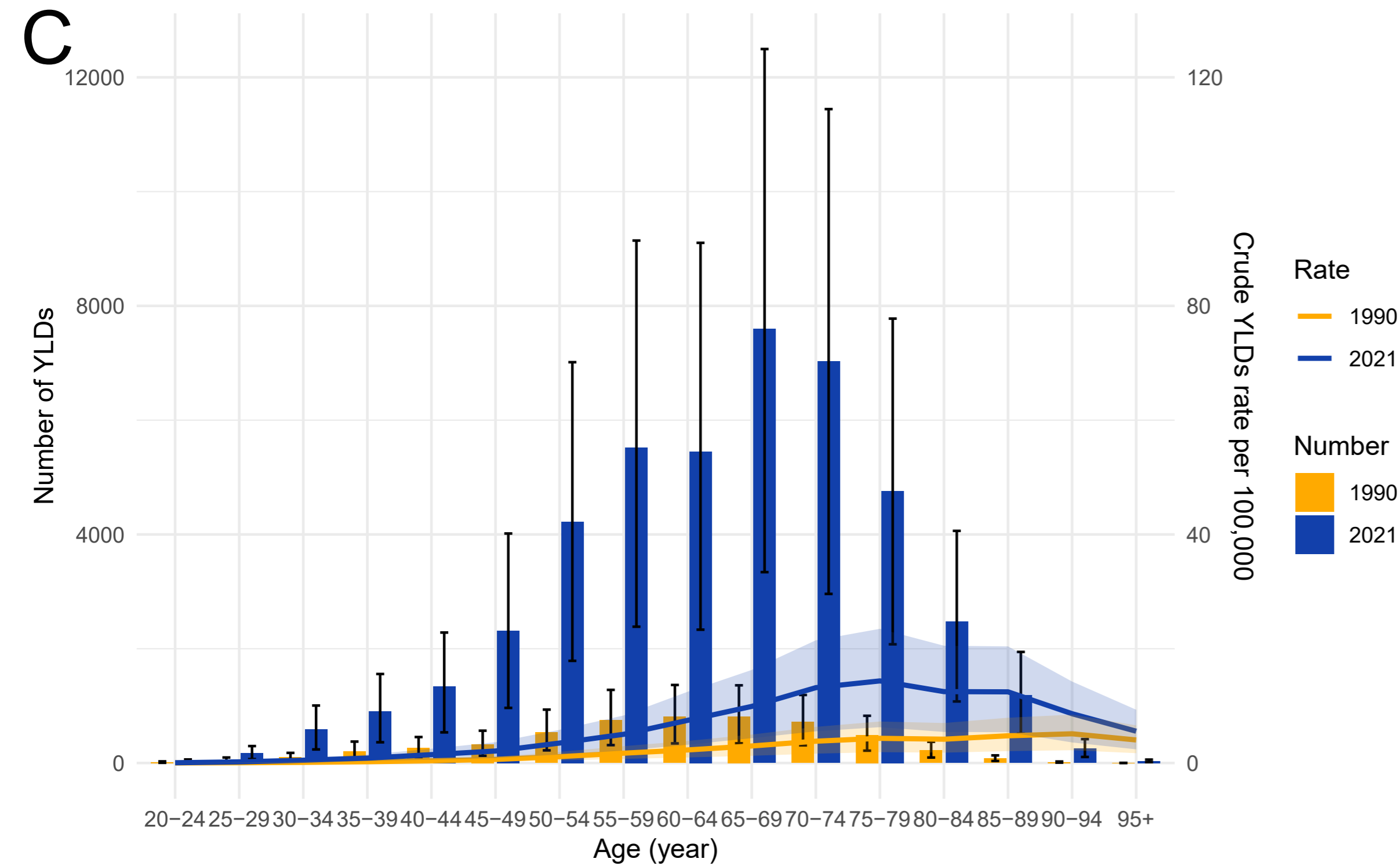

D

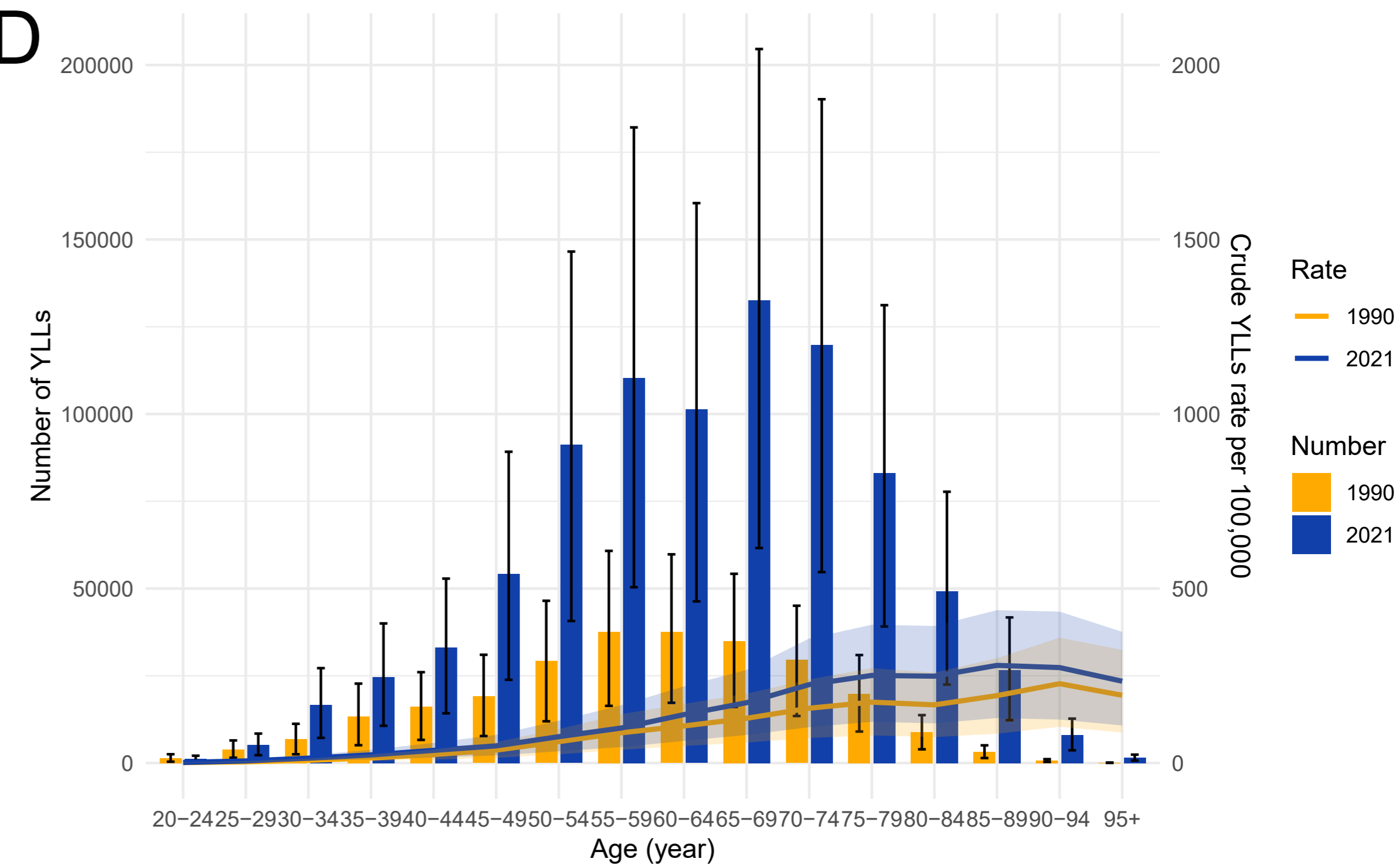

Supplement: Supplementary file 1 [file Image_1.pdf]

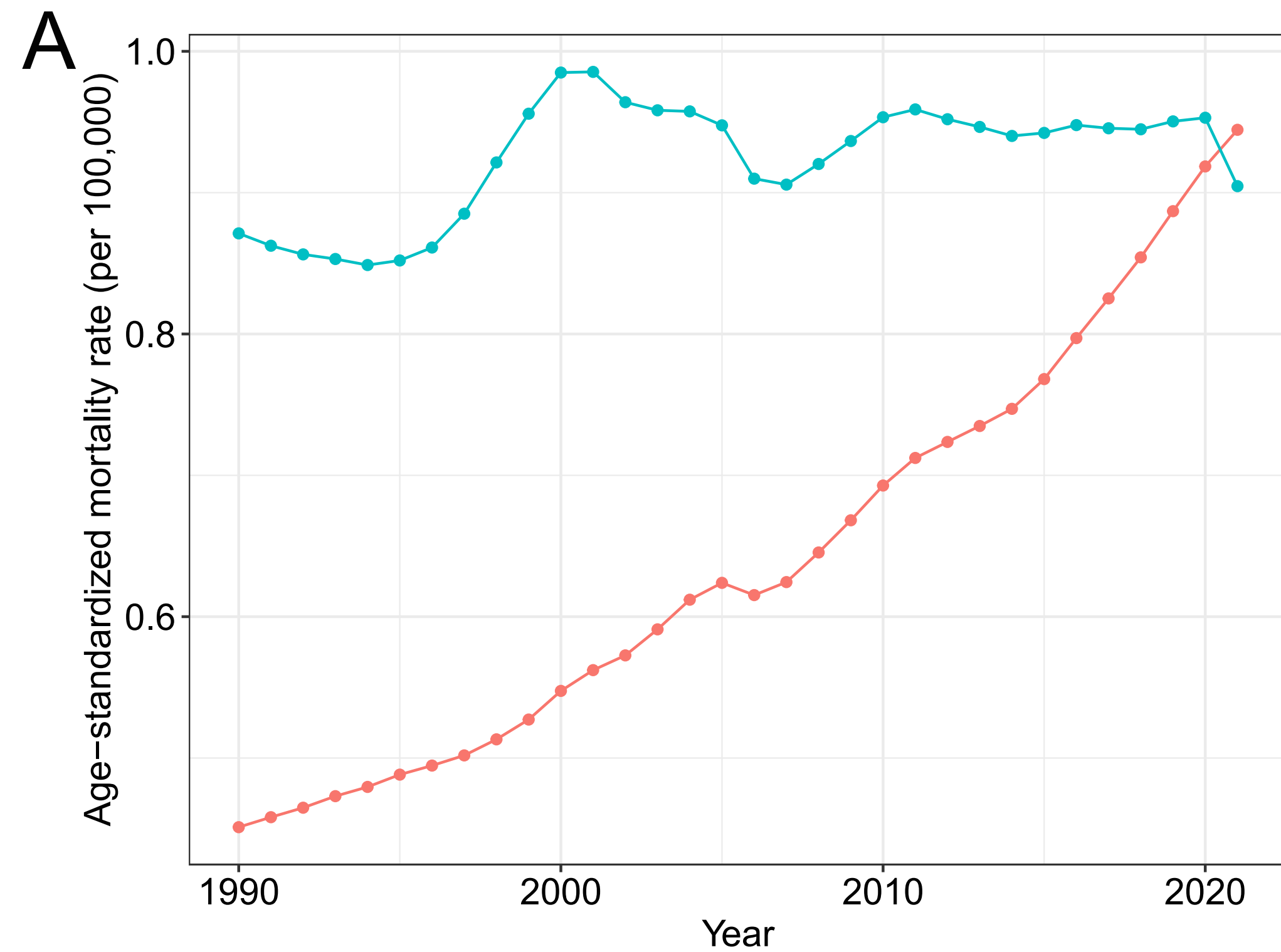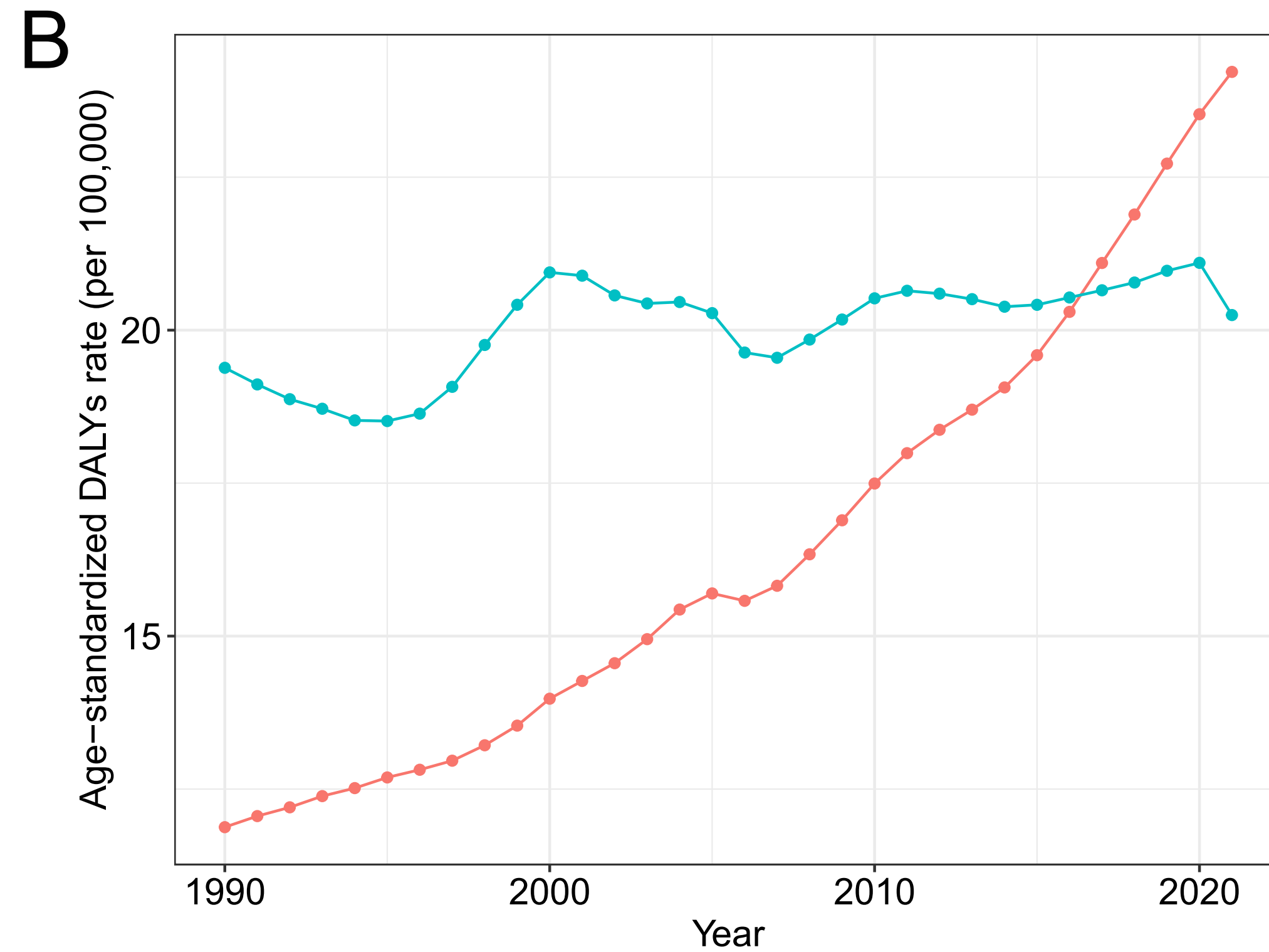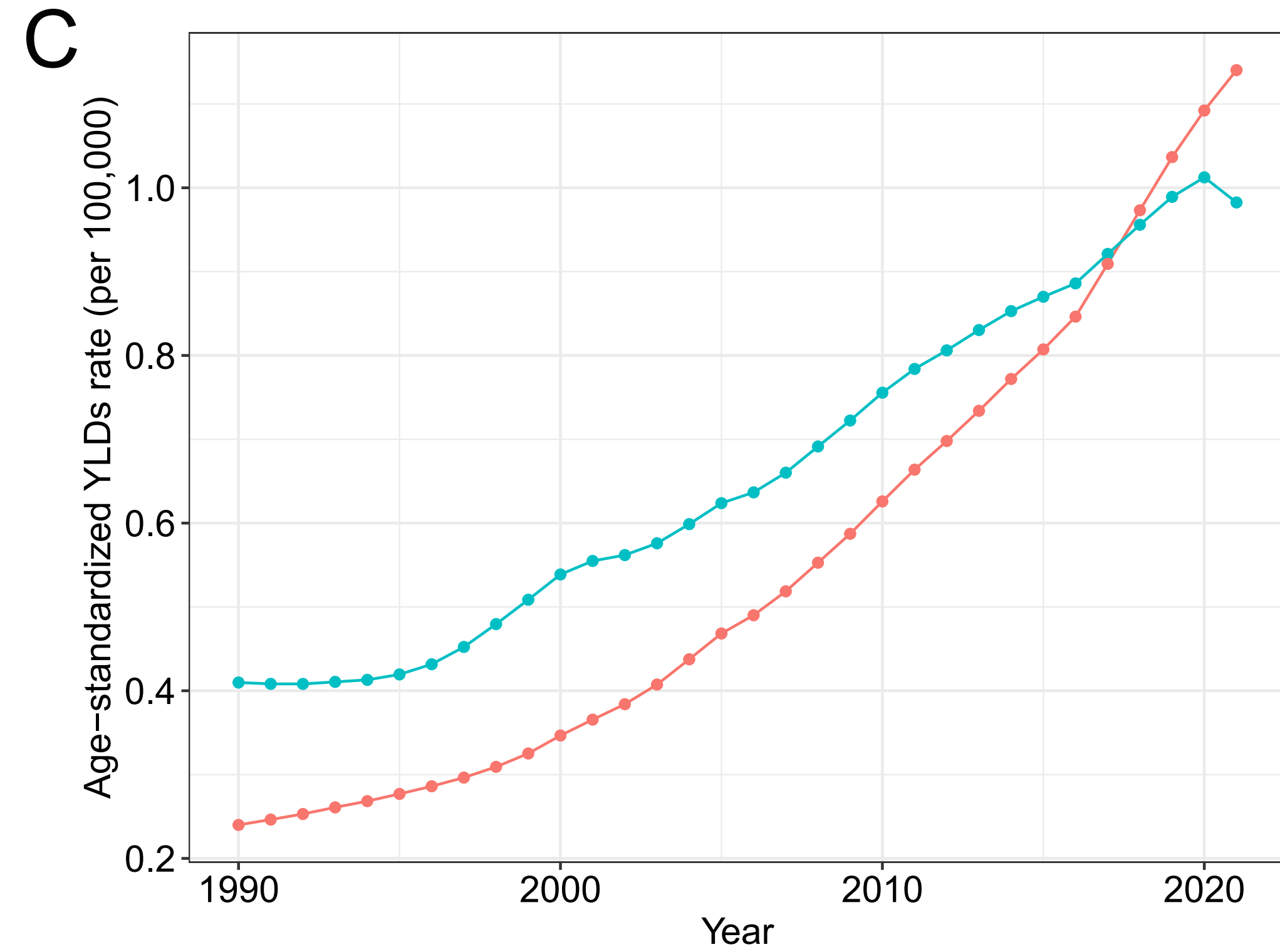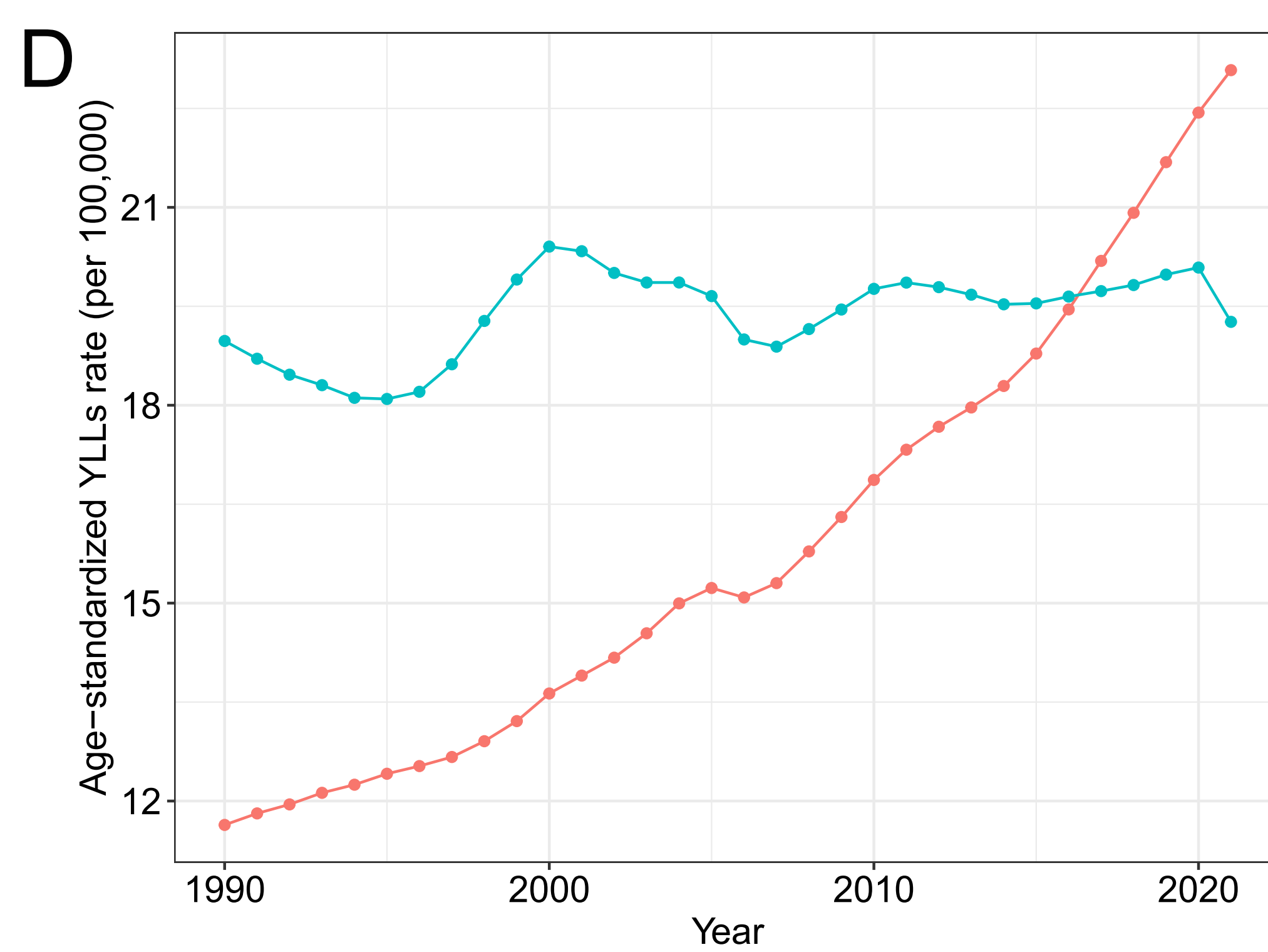

rei\_name

- High body-mass index
- High fasting plasma glucose

Supplement: Supplementary file 2 [file Image_2.pdf]

A

1990–2021 global age-standardized rate

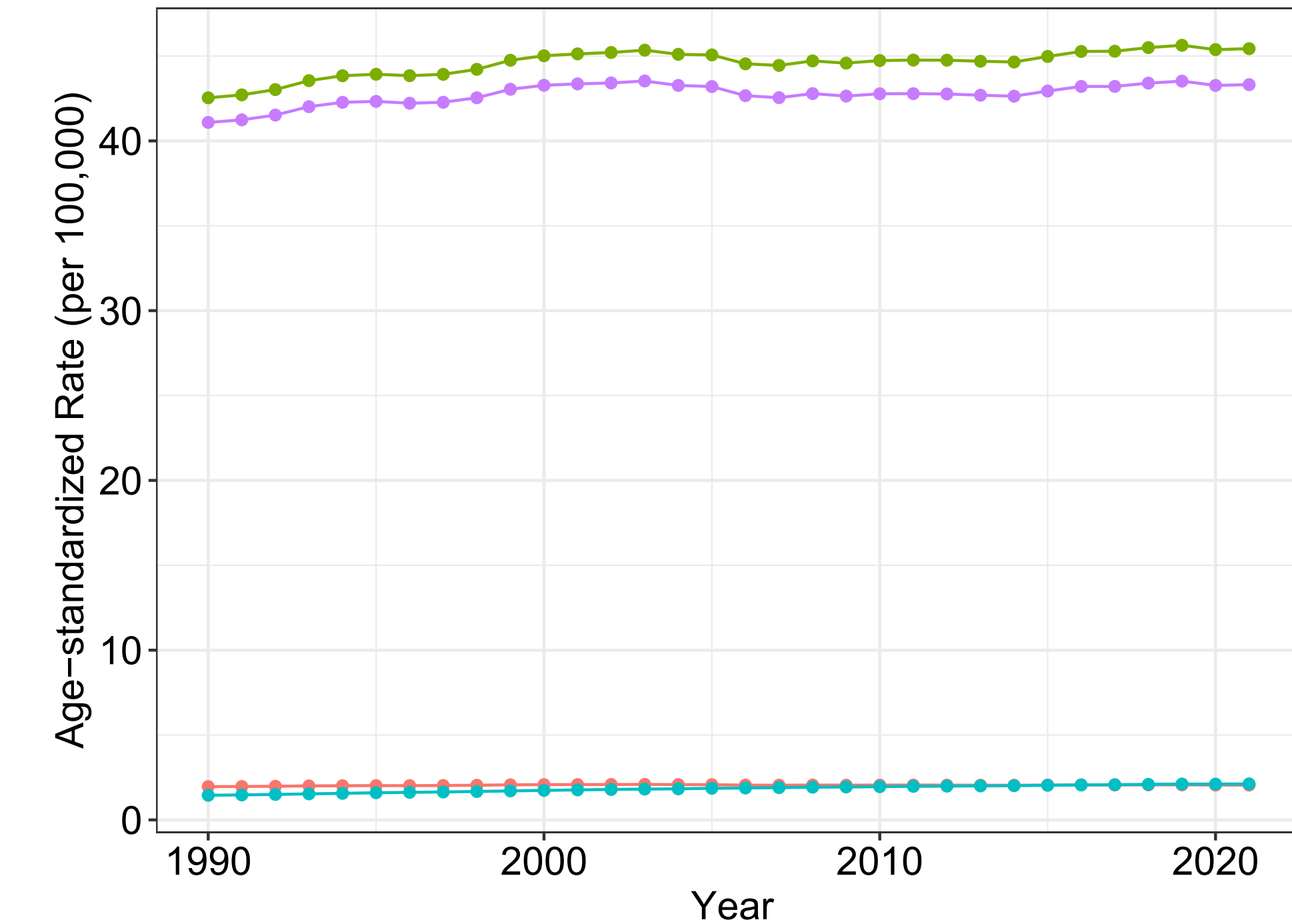

B

1990–2021 China age-standardized rate

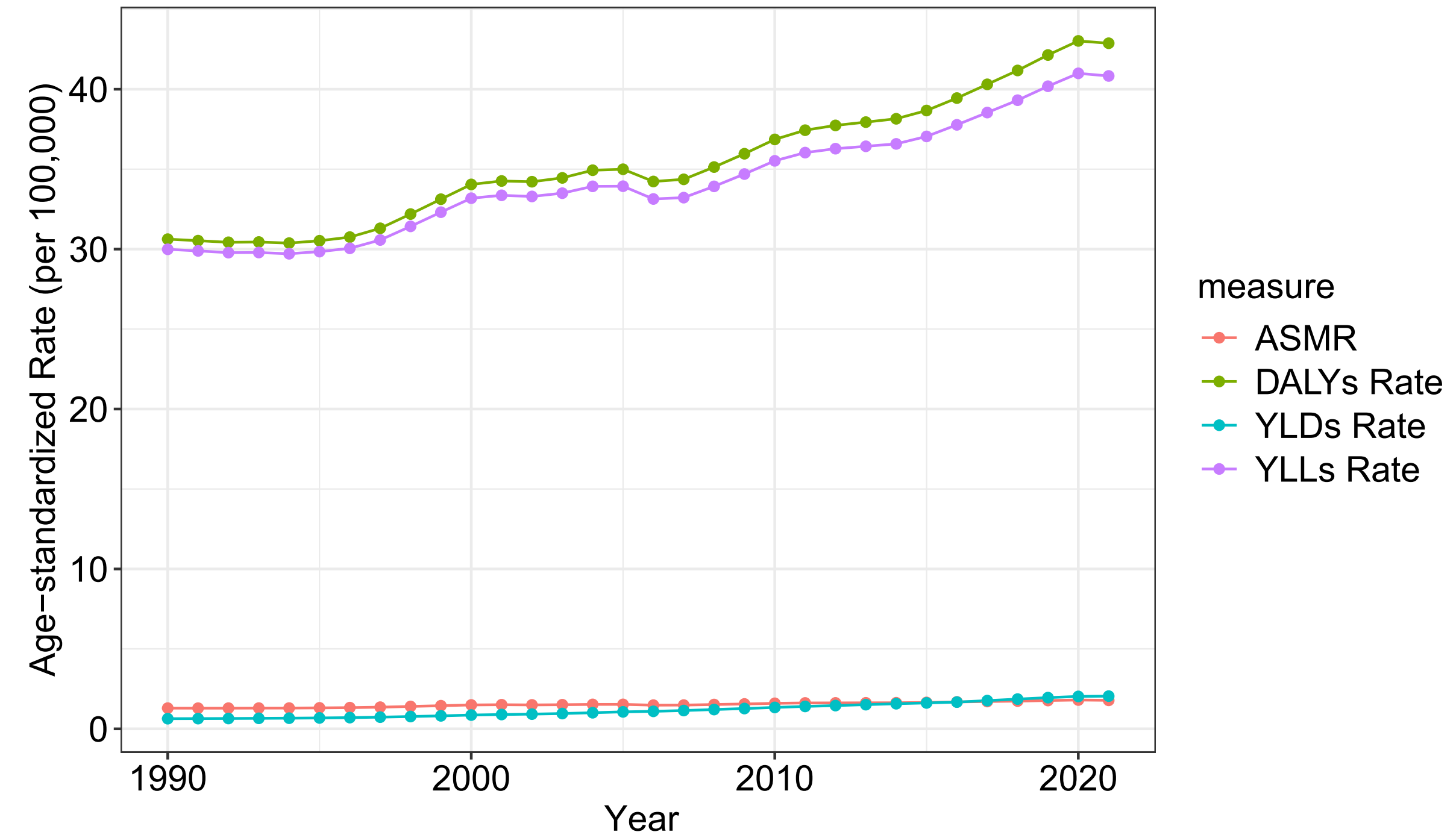

Supplement: Supplementary file 3 [file Image_3.pdf]

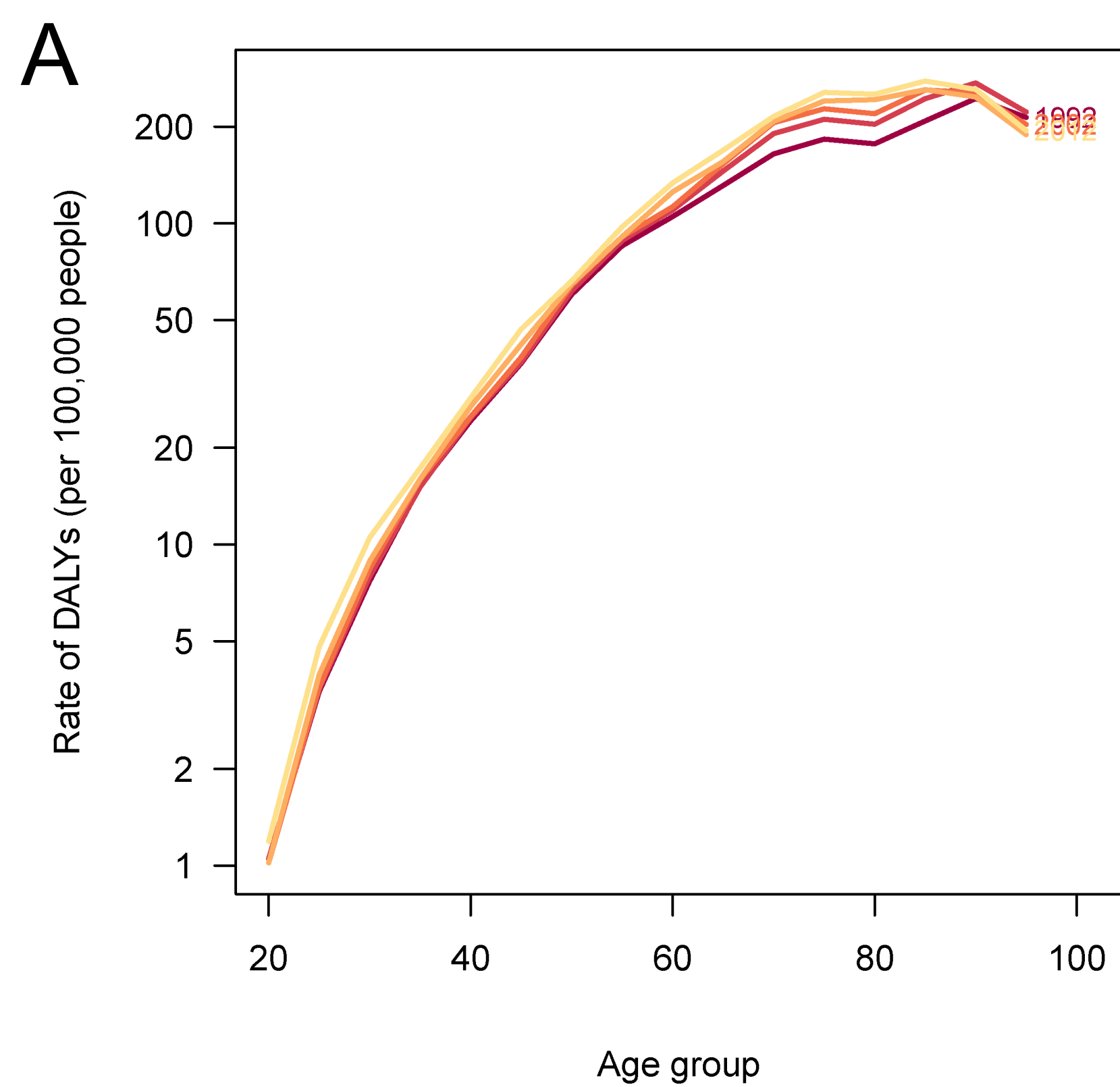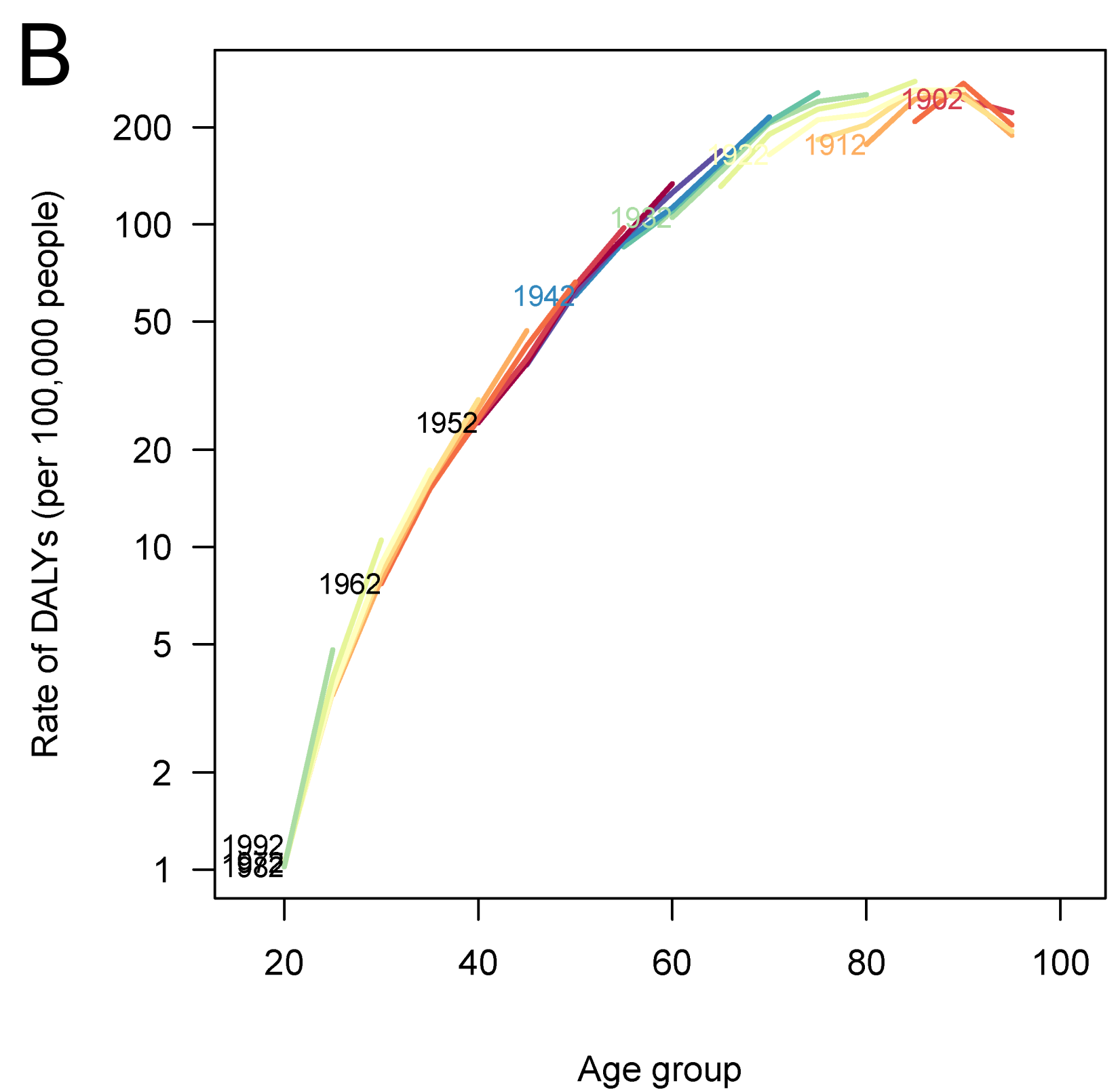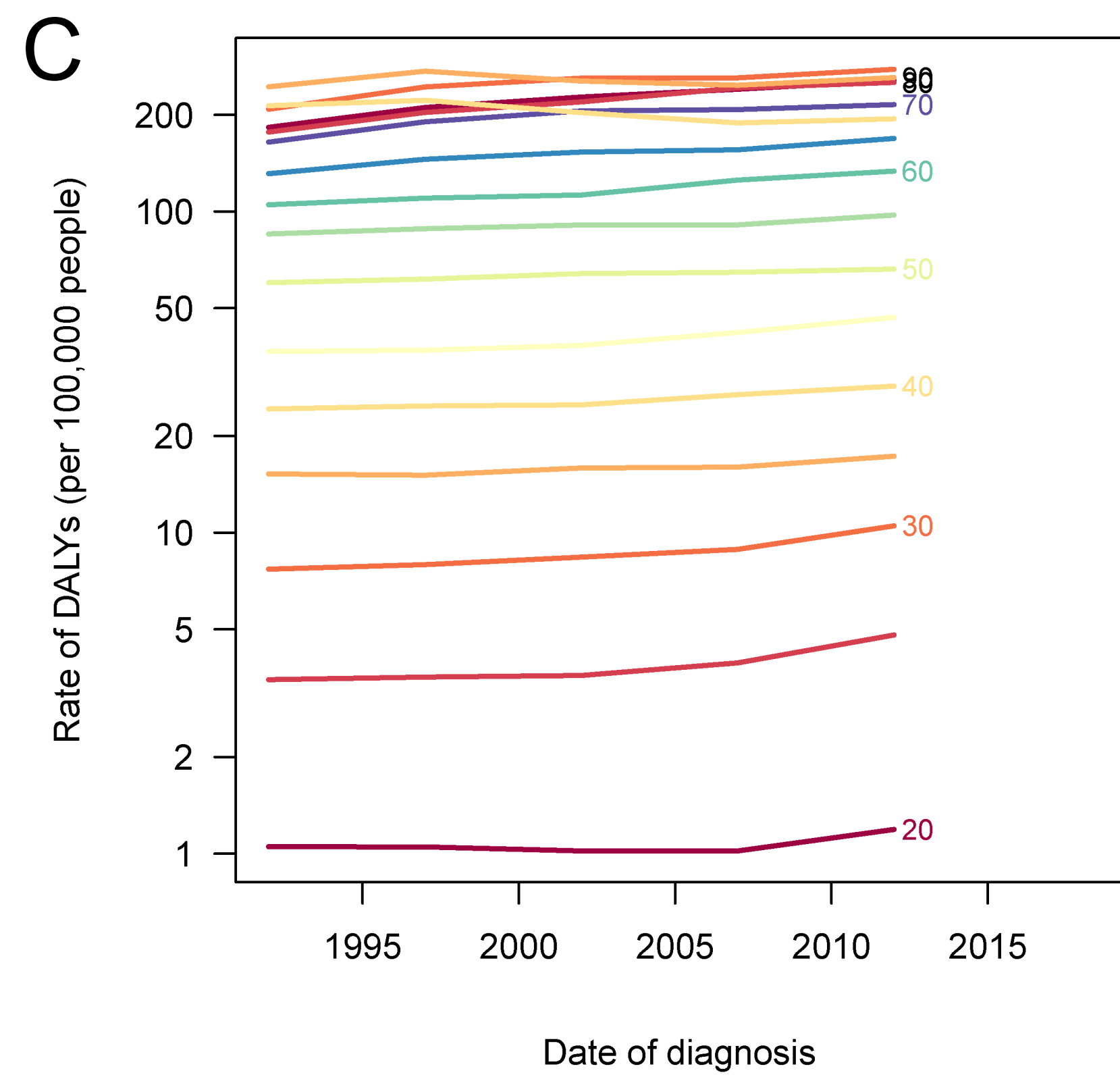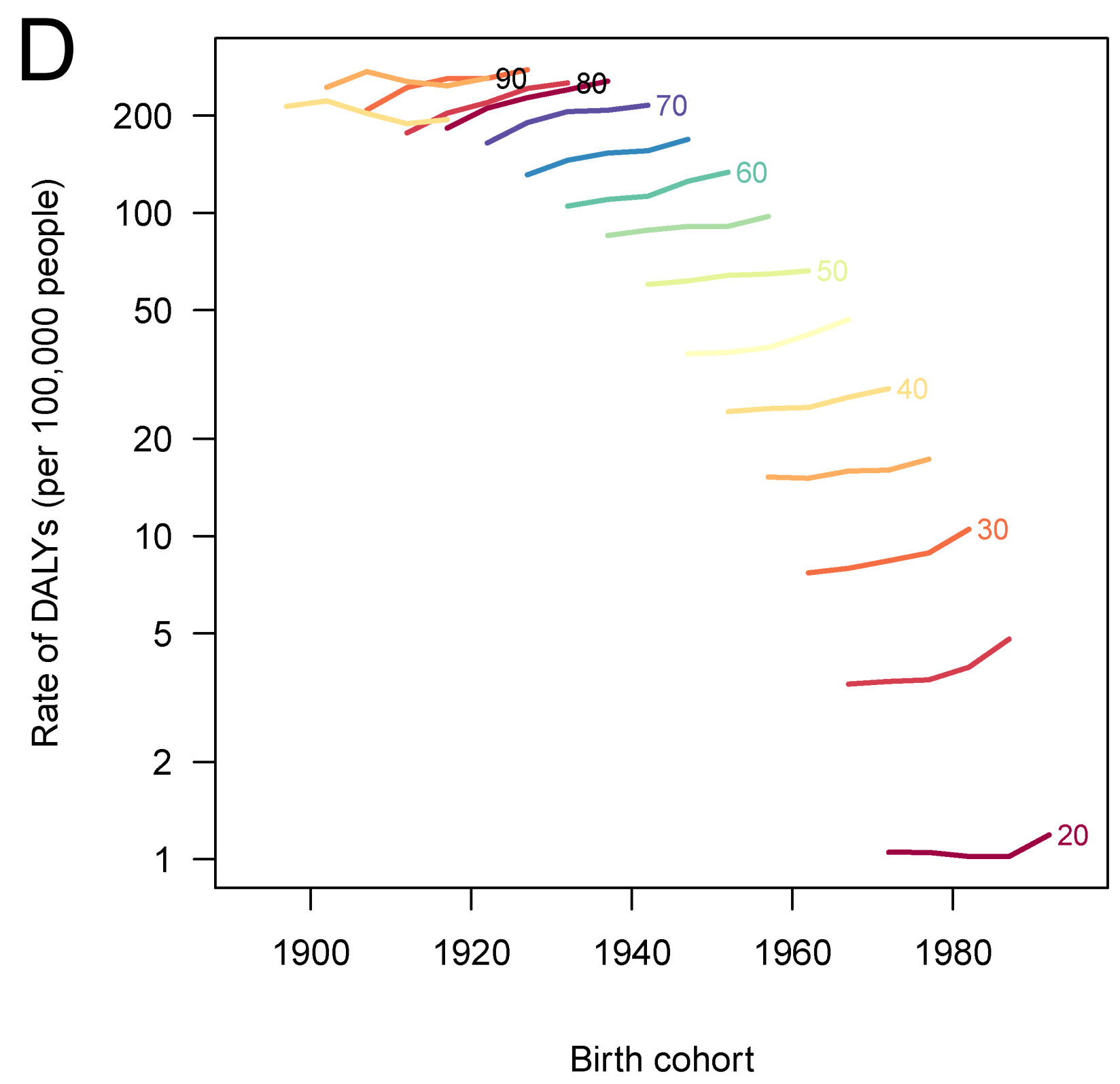

Supplement: Supplementary file 4 [file Image_4.pdf]
