# Supplementary material for: Impact of metabolic risk factors on colorectal cancer burden in China: a comprehensive analysis of trends from 1990 to 2021
Source: Front Nutr. 2026 Jan 8;12:1694231. doi: 10.3389/fnut.2025.1694231 (PMC12824012; doi:10.3389/fnut.2025.1694231)
Supplement: Supplementary file 5 [file Table_1.docx]

Table S1. YLDs and YLLs for CRC attributable to metabolic risks in China, 2021, with trends in ASRs per 100, 000 population, 1990 - 2021

|  | YLDs | | | YLLs | | |
| --- | --- | --- | --- | --- | --- | --- |
| Metabolic risk | No, in thousands | Age-standardized rate per 100, 000 | Percentage change from 1990 to 2021 | No, in thousands | Age-standardized rate per 100, 000 | Percentage change from 1990 to 2021 |
| High body-mass index | 24.4 (9.2, 41.7) | 1.1 (0.4, 2.0) | 375.3 (264.1, 561.7) | 482.9 (200.4, 815.8) | 23.1 (9.6, 38.9) | 98.3 (50.4, 180.1) |
| High fasting plasma glucose | 21.2 (9.7, 35.2) | 1 (0.4, 1.6) | 139.7 (87.1, 209.0) | 408.2 (201.4, 636.5) | 19.3 (9.5, 30.0) | 1.5 (-23.1, 32.3) |

Values in parentheses indicate 95% UIs, estimated using Monte Carlo simulations. Abbreviations: YLDs, years lived with disability; YLLs, years of life lost; ASRs, age-standardized rates; UI, uncertainty interval.
