# Supplementary material for: Impact of metabolic risk factors on colorectal cancer burden in China: a comprehensive analysis of trends from 1990 to 2021
Source: Front Nutr. 2026 Jan 8;12:1694231. doi: 10.3389/fnut.2025.1694231 (PMC12824012; doi:10.3389/fnut.2025.1694231)
Supplement: Supplementary file 6 [file Table_2.docx]

Table S2. Trends in age-standardized mortality, DALY, YLD, and YLL rates (per 100,000 persons) among both sexes, males, and females from 1990 to 2021 for CRC attributable to metabolic risks in China.

|  | Age-standardized mortality rate | | | Age-standardized DALY rate | | | Age-standardized YLD rate | | | Age-standardized YLL rate | | |
| --- | --- | --- | --- | --- | --- | --- | --- | --- | --- | --- | --- | --- |
| Gender | Period | APC (95% CI) | AAPC (95% CI) | Period | APC (95% CI) | AAPC (95% CI) | Period | APC (95% CI) | AAPC (95% CI) | Period | APC (95% CI) | AAPC (95% CI) |
| Both | 1990-1996 | 0.31 (-0.46 - 0.76) | 1.09 (1.02 - 1.15) ^*^ | 1990-1996 | -0.01 (-0.32 - 0.26) | 1.14 (1.10 - 1.19) ^*^ | 1990-1995 | 1.26 (0.76 - 1.81) ^*^ | 3.87 (3.80 - 3.94) ^*^ | 1990-1996 | -0.05 (-0.36 - 0.23) | 1.05 (1.01 - 1.10) ^*^ |
|  | 1996-2000 | 3.37 (2.47 - 4.52) ^*^ |  | 1996-2000 | 2.91 (2.24 - 3.62) ^*^ |  | 1995-2013 | 4.61 (4.10 - 4.73) ^*^ |  | 1996-2000 | 2.85 (2.19 - 3.57) ^*^ |  |
|  | 2000-2007 | 0.11 (-0.90 - 0.49) |  | 2000-2007 | 0.17 (-0.18 - 0.44) |  | 2013-2016 | 3.28 (2.80 - 4.77) ^*^ |  | 2000-2007 | 0.05 (-0.30 - 0.33) |  |
|  | 2007-2021 | 1.26 (1.12 - 1.45) ^*^ |  | 2007-2011 | 2.02 (1.49 - 2.68) ^*^ |  | 2016-2019 | 5.45 (4.22 - 6.12) ^*^ |  | 2007-2011 | 1.90 (1.35 - 2.55) ^*^ |  |
|  |  |  |  | 2011-2014 | 0.56 (0.16 - 1.26) ^*^ |  | 2019-2021 | 2.34 (1.22 - 4.14) ^*^ |  | 2011-2014 | 0.44 (0.04 - 1.15) ^*^ |  |
|  |  |  |  | 2014-2021 | 1.88 (1.70 - 2.30) ^*^ |  |  |  |  | 2014-2021 | 1.77 (1.59 - 2.18) ^*^ |  |
| Female | 1990-1996 | 0.12 (-0.67 - 0.77) | 0.54 (0.48 - 0.60) ^*^ | 1990-1996 | -0.17 (-0.68 - 0.12) | 0.44 (0.38 - 0.49) ^*^ | 1990-1996 | 1.56 (0.87 - 1.99) ^*^ | 3.31 (3.24 - 3.36) ^*^ | 1990-1996 | -0.21 (-0.76 - 0.09) | 0.34 (0.28 - 0.39) ^*^ |
|  | 1996-2000 | 2.50 (-0.20 - 3.36) |  | 1996-2000 | 1.93 (1.20 - 2.63) ^*^ |  | 1996-1999 | 4.90 (1.47 - 5.30) ^*^ |  | 1996-2000 | 1.87 (0.95 - 2.58) ^*^ |  |
|  | 2000-2007 | -0.86 (-1.51 - 2.70) |  | 2000-2007 | -0.84 (-1.34 - -0.25) ^*^ |  | 1999-2010 | 3.43 (3.31 - 4.90) ^*^ |  | 2000-2007 | -0.97 (-1.48 - 0.26) |  |
|  | 2007-2011 | 0.34 (-1.14 - 1.05) |  | 2007-2015 | -0.01 (-0.74 - 0.36) |  | 2010-2016 | 3.02 (2.17 - 3.30) ^*^ |  | 2007-2015 | -0.13 (-0.94 - 0.25) |  |
|  | 2011-2014 | -0.89 (-1.43 - 2.36) |  | 2015-2019 | 2.78 (0.02 - 3.52) ^*^ |  | 2016-2019 | 6.09 (5.29 - 6.68) ^*^ |  | 2015-2019 | 2.66 (-0.17 - 3.42) |  |
|  | 2014-2021 | 1.94 (1.29 - 2.65) ^*^ |  | 2019-2021 | 0.93 (-0.01 - 2.28) |  | 2019-2021 | 2.28 (1.14 - 3.36) ^*^ |  | 2019-2021 | 0.84 (-0.14 - 2.19) |  |
| Male | 1990-1996 | 0.53 (0.07 - 0.85) ^*^ | 1.50 (1.45 - 1.55) ^*^ | 1990-1996 | 0.18 (-0.38 - 0.49) | 1.59 (1.54 - 1.64) ^*^ | 1990-1995 | 1.18 (0.17 - 1.80) ^*^ | 4.29 (4.22 - 4.36) ^*^ | 1990-1996 | 0.15 (-0.39 - 0.46) | 1.50 (1.46 - 1.55) ^*^ |
|  | 1996-2000 | 3.98 (3.35 - 4.86) ^*^ |  | 1996-2000 | 3.42 (1.01 - 4.20) ^*^ |  | 1995-2007 | 5.16 (3.76 - 5.34) ^*^ |  | 1996-2000 | 3.36 (1.48 - 4.16) ^*^ |  |
|  | 2000-2004 | 1.36 (0.80 - 2.24) ^*^ |  | 2000-2004 | 1.54 (0.67 - 2.92) ^*^ |  | 2007-2012 | 5.93 (4.95 - 6.81) ^*^ |  | 2000-2004 | 1.45 (0.56 - 2.75) ^*^ |  |
|  | 2004-2007 | -0.56 (-1.08 - 0.28) |  | 2004-2007 | -0.03 (-0.52 - 3.40) |  | 2012-2021 | 3.99 (3.64 - 4.26) ^*^ |  | 2004-2007 | -0.18 (-0.67 - 3.23) |  |
|  | 2007-2010 | 3.70 (2.72 - 4.18) ^*^ |  | 2007-2010 | 3.73 (1.43 - 4.16) ^*^ |  |  |  |  | 2007-2010 | 3.62 (1.45 - 4.06) ^*^ |  |
|  | 2010-2021 | 1.17 (1.01 - 1.29) ^*^ |  | 2010-2021 | 1.60 (1.44 - 1.79) ^*^ |  |  |  |  | 2010-2021 | 1.49 (1.33 - 1.64) ^*^ |  |

Abbreviations: CRC, colon and rectum cancer; DALYs, disability-adjusted life-years; YLDs, years lived with disability; YLLs, years of life lost; AAPC, average annual percent change presented for full period; APC, annual percent change; CI, confidence interval. ^*^, *p* <0.05.
